# Additional copies of 1q negatively impact the outcome of multiple myeloma patients and induce transcriptomic deregulation in malignant plasma cells

**DOI:** 10.1038/s41408-024-01075-x

**Published:** 2024-06-07

**Authors:** Mattia D’Agostino, Delia Rota-Scalabrini, Angelo Belotti, Luca Bertamini, Maddalena Arigoni, Giovanni De Sabbata, Giuseppe Pietrantuono, Anna Pascarella, Patrizia Tosi, Francesco Pisani, Norbert Pescosta, Marina Ruggeri, Jennifer Rogers, Martina Olivero, Mariagrazia Garzia, Piero Galieni, Ombretta Annibali, Federico Monaco, Anna Marina Liberati, Salvatore Palmieri, Paola Stefanoni, Elena Zamagni, Benedetto Bruno, Raffaele Adolfo Calogero, Mario Boccadoro, Pellegrino Musto, Francesca Gay

**Affiliations:** 1 Division of Hematology, AOU Città della Salute e della Scienza di Torino, University of Torino and Department of Molecular Biotechnology and Health Sciences, Torino, Italy; 2 Medical Oncology Department Candiolo Cancer Institute, FPO-IRCCS, Torino, Italy; 3 Department of Hematology, ASST Spedali Civili di Brescia, Brescia, Italy; 4 Department of Hematology, Erasmus MC Cancer institute Rotterdam, Rotterdam, the Netherlands; 5 BGcore, Department of Molecular Biotechnology and Health Sciences, University of Torino, Torino, Italy; 6 Ematologia, Azienda Sanitaria Universitaria Giuliano Isontina, Trieste, Italy; 7 Hematology and Stem Cell Transplantation Unit, IRCCS Centro di Riferimento Oncologico della Basilicata, Rionero in Vulture, Italy; 8 UO Ematologia, Ospedale dell’Angelo, Venezia Mestre, Italy; 9 UO Ematologia, Ospedale di Rimini, Rimini, Italy; 10 IRCCS Istituto Nazionale dei Tumori Regina Elena, Rome, Italy; 11 Ospedale Provinciale Bolzano, Reparto Ematologia e Centro TMO, Bolzano, Italy; 12 Department of Molecular Biotechnology and Health Sciences, University of Torino, Torino, Italy; 13 Multiple Myeloma Research Foundation (MMRF), Norwalk, CT USA; 14 Department of Oncology, University of Torino, Torino, Italy; 15 Hematology and Stem Cell Transplant Unit, Az. Osp. San Camillo Forlanini, Rome, Italy; 16 UOC Ematologia e Terapia cellulare, Ospedale C. e G. Mazzoni, Ascoli Piceno, Italy; 17 Hematology, stem cell transplantation, Fondazione Policlinico Universitario Campus Bio medico di Roma, Rome, Italy; 18 SCDU Ematologia, Azienda Ospedaliera SS. Antonio e Biagio e Cesare Arrigo, Alessandria, Italy; 19 S.C. di Oncoematologia, AO Santa Maria di Terni/ Università degli studi di Perugia, Terni-Perugia, Italy; 20 Division of Hematology, Cardarelli Hospital, Naples, Italy; 21 Division of Hematology, ASST Papa Giovanni XXIII, Bergamo, Italy; 22 IRCCS Azienda Ospedaliero-Universitaria di Bologna, Istituto di Ematologia “Seràgnoli”, Dipartimento di Medicina Specialistica, Diagnostica e Sperimentale, Università di Bologna, Bologna, Italy; 23 European Myeloma Network (EMN), Torino, Italy; 24 Department of Precision and Regenerative Medicine and Ionian Area, “Aldo Moro” University School of Medicine, Bari, Italy; 25 Hematology and Stem Cell Transplantation Unit, AOU Consorziale Policlinico, Bari, Italy

**Keywords:** Risk factors, Myeloma, Myeloma

## Abstract

Additional copies of chromosome 1 long arm (1q) are frequently found in multiple myeloma (MM) and predict high-risk disease. Available data suggest a different outcome and biology of patients with amplification (Amp1q, ≥4 copies of 1q) vs. gain (Gain1q, 3 copies of 1q) of 1q. We evaluated the impact of Amp1q/Gain1q on the outcome of newly diagnosed MM patients enrolled in the FORTE trial (NCT02203643). Among 400 patients with available 1q data, 52 (13%) had Amp1q and 129 (32%) Gain1q. After a median follow-up of 62 months, median progression-free survival (PFS) was 21.2 months in the Amp1q group, 54.9 months in Gain1q, and not reached (NR) in Normal 1q. PFS was significantly hampered by the presence of Amp1q (HR 3.34 vs. Normal 1q, *P* < 0.0001; HR 1.99 vs. Gain1q, *P* = 0.0008). Patients with Gain1q had also a significantly shorter PFS compared with Normal 1q (HR 1.68, *P* = 0.0031). Concomitant poor prognostic factors or the failure to achieve MRD negativity predicted a median PFS < 12 months in Amp1q patients. Carfilzomib–lenalidomide–dexamethasone plus autologous stem cell transplantation treatment improved the adverse effect of Gain1q but not Amp1q. Transcriptomic data showed that additional 1q copies were associated with deregulation in apoptosis signaling, p38 MAPK signaling, and Myc-related genes.

## Introduction

Multiple myeloma (MM) is a common hematological neoplasia, and in the last 2 decades, a meaningful survival improvement has been reached thanks to the introduction of novel drugs, with nowadays more than 60% of transplant-eligible patients alive 8 years from initial diagnosis [1]. Nevertheless, despite this improvement, there is still a subset of patients with a reduced benefit from new treatment approaches and a dismal outcome. These patients with “high risk” disease represent a very heterogeneous group, including patients with high tumor burden, genomic and cytogenetic alterations, gene expression profiles, or presence of circulating tumor cells and extramedullary disease [2].

The genomic and cytogenetic events that occur in the genesis, evolution, and progression of MM have been thoroughly investigated [3, 4]. Recurrent chromosomal abnormalities that can be detected by interphase fluorescent in situ hybridization (iFISH) allowed a classification of MM patients based on cytogenetic events, with del(17p), t(4;14) and t(14;16) classically considered as high-risk by IMWG [5].

Indeed, additional copies of chromosome 1q are one of the most frequent chromosomal abnormalities, reported in precursor stages, found in 30–40% of newly diagnosed (ND) MM patients [6] and 50–80% of relapsed-refractory patients [7]. The clonal size [8] of 1q copies seems to correlate with the prognosis. Moreover, the prognostic value of additional copies of 1q21 may depend on the presence of other primary genetic events [9]. Several reports, including clinical trials and real-world data, correlate the presence of 1q abnormalities with adverse outcomes [10–16]. Based on these data, 1q alterations have been incorporated in recent staging systems for NDMM [17–19]. As an example, 1q21+ has recently been included in the R2-ISS [18], and other analyses investigated the prognostic impact of Amp1q (at least four copies of 1q) vs. Gain1q (three copies of 1q) [15, 19–21].

The possible role of specific novel treatment approaches in reducing the adverse outcomes related to high-risk features is currently under evaluation. Response rates are similar in patients with or without 1q alterations, but progression-free survival (PFS) and overall survival (OS) are inferior in patients with 1q abnormalities vs not if patients are treated with autologous stem cell transplantation (ASCT) [15], immunomodulatory drugs (IMID) or bortezomib [14, 21].

Few data are available yet on the impact of Carfilzomib in transplant-eligible NDMM patients with 1q abnormalities.

Hyperexpression of genes located in the 1q region has also been associated with adverse prognosis [22]. The adverse outcome related to 1q copy number and 1q clonal size suggests a “dose effect”, where several genes located on chromosome 1q could be involved (e.g. CKS1B, PSMD4, ADAR1, MCL-1); nevertheless, it is not clear if 1q alteration could mirror genetic instability rather than be the real driver of poor prognosis [23]. The identification of candidate key genes and pathways that are behind 1q abnormalities could potentially pave the way for new therapeutic targets.

The primary aim of our analysis is to evaluate the impact of Amp1q vs Gain1q on outcomes in patients enrolled in the randomized FORTE clinical trial (NCT02203643) in order to describe their impact in a carfilzomib-based therapy setting on the background of the established role of Gain/Amp1q as a broad prognostic factor. Our secondary aim is to understand the transcriptomic changes in Amp1q, Gain1q, and Normal 1q that could elucidate the biological drivers of the adverse outcome related to Amp1q/Gain1q.

## Methods

### Study design and participants

ND transplant-eligible MM patients aged 65 years or younger were enrolled between February 23, 2015 and April 5, 2017. Patients were randomized (R1) into three induction/intensification/consolidation arms: the KCd-ASCT arm included four 28-day induction cycles with KCd, melphalan 200 mg/sqm followed by ASCT (MEL200-ASCT), and four KCd consolidation cycles. the KRd12 arm consisted of twelve 28-day cycles with KRd without upfront MEL200-ASCT; Patients in the KRd-ASCT arm received four 28-day induction cycles with KRd, MEL200-ASCT, and four KRd consolidation cycles.

After consolidation, a second randomization (R2) to two maintenance regimens was performed in eligible patients. Patients in the KR maintenance arm received carfilzomib for up to 2 years and lenalidomide until progression, while patients in the R maintenance arm received lenalidomide alone until progression. Full details on the study treatment were previously described [24].

The FORTE trial was approved by the ethics or Institutional Review Boards at each of the participating centers. All patients gave written informed consent before entering the study, which was performed in accordance with the Declaration of Helsinki and the Good Clinical Practice guidelines.

### Cytogenetic risk evaluation

Cytogenetic risk was assessed by a centralized laboratory receiving bone marrow aspirate samples collected during the screening phase. Chromosome abnormalities (CA) were assessed by FISH analysis performed on CD138+ purified bone marrow plasma cells (BMPCs). BMPCs were enriched using anti-CD138-coated magnetic microbeads and an AutoMACS Pro separator (Miltenyi Biotech) following the manufacturer’s instructions [25]. The FISH analysis included probes to detect conventionally defined high-risk disease [t(4;14) and/or t(14;16) and/or del(17p)] [26] and Gain/Amp1q as well (LPH039 probe, Cytocell™, Oxford Gene Technology, Oxford, England).

Two hundred BMPC nuclei from each sample were counted and scored. The cut-off value for CA positivity was 15% for translocations and 10% for copy number alterations. Cut-offs were defined according to the mean ± 3 standard deviations of abnormal signals detected in the bone marrow plasma cells of healthy donors [25, 27].

Patients were defined as Gain(1q) positive if ≥10% of nuclei with ≥3 copies of 1q were detected and the definition of Amp(1q) was not met.

Patients were defined as Amp(1q) positive if ≥20% of nuclei with ≥4 copies of 1q21 were detected. The 20% cut-off for Amp(1q) positivity was recommended by FISH guidelines [27] and consistent with the one applied by other groups [14, 28]. The reliability of the cut-off was further verified using an Epanechnikov kernel-smoothed estimated hazard rate to study the risk of progression or death according to the % of nuclei with ≥4 copies of 1q (Fig. S1).

### MRD evaluation

A centralized evaluation of minimal residual disease (MRD) by multiparameter flow cytometry (MFC; sensitivity threshold of 10-5) [24] was performed before maintenance and thereafter every 6 months during maintenance in all patients achieving at least a very good partial response (VGPR). Data were analyzed on intention-to-treat (ITT), with patients with a positive MRD test result or who had not undergone MRD testing (either because they missed evaluation, or they achieved < VGPR) considered MRD positive. The calculation of 1-year sustained MRD negativity is described in the supplementary appendix.

### RNA sequencing data generation and analysis

Data from patients enrolled in the prospective observational Multiple Myeloma Research Foundation (MMRF) CoMMpass study (NCT01454297) were used as a test set, and data from additionally sequenced patients enrolled in the FORTE trial were used as a validation set.

The Interim Analysis (IA)14 release of CoMMpass was used for the analyses, 1q copy number abnormalities (CNAs) in the CoMMpass patients were defined using molecular data with a technique (SeqFISH) that was previously cross-validated against conventional FISH [29]. RNAseq data in the CoMMpass study were generated as previously described [30].

Data deposition, RNAseq data analysis, and RNAseq data generation in the additionally sequenced patients enrolled in the FORTE trial are described in the Supplementary appendix.

### Statistical analysis

The efficacy analysis included patients in the ITT population who met the biomarker criteria for risk assessment (1q probe analyzed by FISH and number of 1q copies available), whereas patients with incomplete data about 1q copy number were excluded from this analysis. Patients with evaluable 1q copy numbers were stratified into three groups according to the presence of Amp(1q), Gain(1q), or no 1q abnormalities (Normal 1q) as defined above. The aim of this analysis was to compare the outcome of patients with Amp(1q), Gain(1q), and Normal 1q.

This analysis was a post-hoc analysis that was not prespecified in the protocol.

PFS was calculated from the date of randomization (R1 or R2, according to the endpoint) to the date of first observation of progressive disease (PD) or death from any cause. Patients who did not progress or were lost to follow-up, or who withdrew from the trial were censored at the time of the last disease assessment.

OS was calculated from the date of first randomization to the date of death from any cause.

Time-to-event data were analyzed using the Kaplan–Meier method. The Cox proportional hazards model was used to estimate the HR values and the 95% confidence intervals (CIs). Cox models were adjusted for Revised-International Staging System (R-ISS) stage (R-ISS III vs. II vs. I vs. not available), age (≥60 vs. <60 years), and randomization arm (KRd-ASCT vs. KRd12 vs. KCd-ASCT). The significance level was set at *P* < 0·05.

The statistical analyses were performed using R (v4.0·2). The data cut-off point was January 7, 2021.

### Role of the funding source

The UNITO-MM-01/FORTE trial was sponsored by the Università degli Studi di Torino (Turin, Italy), Department of Molecular Biotechnology and Health Sciences. Amgen and Celgene/Bristol Myers Squibb provided an unrestricted grant to conduct the trial but had no role in study design, data collection, data analysis, data interpretation, writing of the report, or the decision to submit the manuscript for publication. International Myeloma Society (IMS) supported the research analyses presented in this paper with an award conferred to the principal investigator of the trial.

## Results

### Study population

**Fig. 1:**
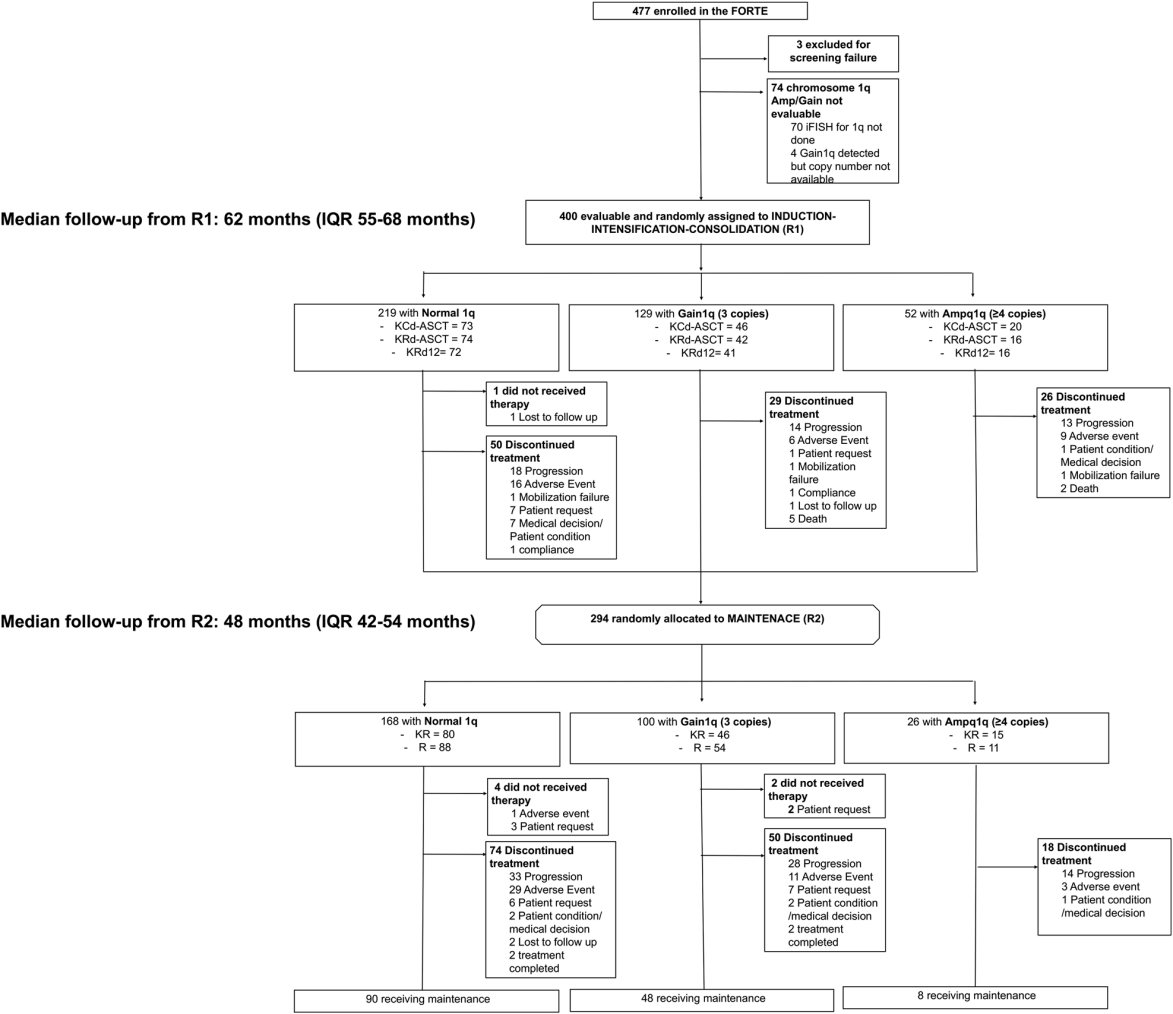
Consort diagram of patients enrolled in the FORTE trial.

A total of 477 patients were enrolled in the FORTE trial and 474 were randomized into one of the three induction–intensification–consolidation arms (R1). In 74 patients, the evaluation of 1q abnormalities was missing, or 1q copy number was not available (Fig. 1). Of 400 patients evaluable for 1q abnormalities (ITT 1q population), 52 (13%) had Amp1q, 129 (32%) Gain1q and 219 (55%) had Normal 1q.

Regarding group disposition, 26 patients did not meet the definition of Amp1q but had a borderline number of nuclei with ≥4 copies of 1q (10–19% of total cells). All these 26 patients were not classified as Amp1q, but they were classified as Gain1q because they had a mean number of nuclei with three copies of 59% (range 33–78%) and a mean number of nuclei with ≥3 copies (3 copies + ≥4 copies) of 73% (range 48–91%).

Median follow-up from the first randomization was 62 months [Interquartile range (IQR) 55–68 months].

Demographic features and treatment were well balanced between patients with Amp1q, Gain1q, and Normal1q. High-risk disease features (ISS stage II–III, high-risk cytogenetics, and high LDH) were enriched in the group of patients with 1q abnormalities compared to Normal 1q with no statistically significant differences between patients with Amp1q vs. Gain1q, except for an increased proportion of patients with high LDH (*p* = 0.0024) in Amp1q group (Table 1). Of note, patients with hyperdiploid status were less represented in Amp1q cases (23% vs. 36% vs. 45% in Amp1q vs. Gain1q vs. Normal1q cases, *p* = 0.016).

**Table 1:** Patient characteristics of patients randomized for induction therapy (R1) in the FORTE trial according to 1q21 status.

		FORTE population	1q Amp/gain not evaluable^a^	Amp1q	Gain1q	Normal 1q	*p*-value^b^
		*N* = 474	*N* = 74	*N* = 52	*N* = 129	*N* = 219	
Age	Median (IQR)	57- (51–62)	55- (50–61)	59 – (53–62)	58 – (53–63)	57- (51–62)	0.6693
ISS	I	240 (51)	50 (68)	15 (29)	61 (47)	114 (52)	0.0764
	II	152 (32)	15 (20)	23 (44)	41 (33)	73 (33)	
	III	82 (17)	9 (12)	14 (27)	27 (15)	32 (15)	
Cytogenetic abnormalities	Standard risk	275 (67)	5 (56)	25 (48)	74 (57)	171 (78)	0.3223
	High risk^c^	133 (33)	4 (44)	27 (52)	55 (43)	47 (22)	
	Missing	66	65	0	0	1	
	del(17p)	58 (15)	0	11 (21)	15 (12)	32 (15)	0.1064
	t(4;14)	65 (16)	0	17 (33)	31 (24)	17 (8)	0.2656
	t(14;16)	21 (5)	2 (3)	7 (13)	11 (9)	2 (1)	0.4097
LDH	Normal	397 (87)	68 (93)	31 (65)	108 (86)	190 (90)	0.0024
	High	61 (13)	5 (7)	17 (35)	17 (14)	22 (10)	
	Missing	16	1	4	4	7	
Induction Therapy (R1)	KCd-ASCT	159 (34)	20 (27)	20 (38)	46 (36)	73 (33)	0.9360
	KRd-ASCT	157 (33)	28 (38)	16 (31)	41 (32)	72 (33)	
	KRd-12	158 (33)	26 (35)	16 (31)	42 (33)	74 (34)	

Percentage may not total 100 because of rounding. % calculated on the available patients.

*IQR* interquartile range, *ISS* International Staging System, *LDH* lactate dehydrogenase; *FISH* fluorescence in situ hybridization, *K* carfilzomib, *R* lenalidomide, *d* dexamethasone, *KCd-ASCT*: KCd induction-ASCT-KCd consolidation, *KRd-ASCT* KRd induction-ASCT-KRd consolidation, *KRd12* 12 cycles of KRd.

^a^70/474: 1q CNA not done, 4/474: Gain1q detected but copy number not available.

^b^Amp1q vs. Gain1q comparison.

^c^High risk-cytogenetic abnormalities: defined as del(17p) and/or t(4;14) and/or t(14;16).

### ITT analysis

In the ITT analysis, rate of premaintenance ≥ VGPR was 77% (95% CI 63–87%) in Amp1q, 84% (95% CI 76–90%) in Gain1q and 84% (95% CI 78–88%) in Normal 1q (*p* = 0.49); premaintenance ≥ CR was achieved in 46% (95% CI 32–61%), 47% (95% CI 38–55%) and 52% (95% CI 45–58%) of patients, respectively (*p* = 0.59). Similarly, the rate of premaintenance 10^−5^ MRD negativity MFC was comparable in Amp1q [44% (95% CI 30–59%)], Gain1q [55% (95% CI 46–64%)], and Normal 1q [55% (95% CI 48–62%)] (*p* = 0.35). There was a trend towards a lower 1-year sustained MRD negativity rate in Amp1q [19% (95% CI 10–33%)] vs Gain1q [32% (95% CI 24–41%)] and Normal 1q [37% (95% CI 31–44%)] (*p* = 0.05) (Table S1).

**Fig. 2:**
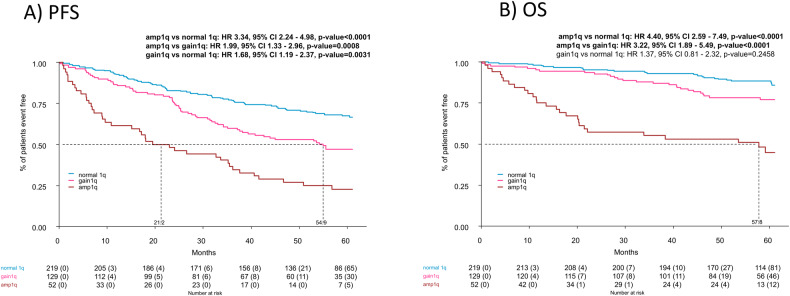
Outcomes according to 1q copies. Progression-free survival (**A**) and overall-survival (**B**) from first randomization (R1) according to 1q subgroups. HR values and the 95% CIs were estimated with a Cox proportional hazards model adjusted for the R-ISS stage (R-ISS III vs. II vs. I vs. not available), age (≥60 vs. <60 years), and randomization arm (KRd-ASCT vs. KRd12 vs. KCd-ASCT).

PFS from R1 (Fig. 2A) was significantly inferior in the presence of Amp1q vs. Normal1q (HR 3.34, 95% CI 2.24–4.98, *p* < 0.0001) and Amp1q vs. Gain1q (HR 1.99, 95% CI 1.33–2.96, *p* = 0.0008). Patients with Gain1q had also a significantly shorter PFS compared with Normal 1q (HR 1.68, 95% CI 1.19–2.37, *p* = 0.0031). 4-year PFS rate was 27% (95% CI 17–42%)in Amp1q, 53% (95% CI 45–63%) in Gain1q and 71% (95% CI 65–77%) in Normal 1q.

We ran an additional analysis using a more conservative cut-off to define Gain1q (20% instead of 10%). Only 19 patients were reclassified from Gain1q positive to Normal 1q, and the results were comparable to what was observed in the main analysis using a 10% cut-off (Supplementary appendix).

The presence of Amp1q was also associated with significantly shorter OS compared to Normal 1q (HR 4.40, 95% CI 2.59–7.49, *p* < 0.0001) and to Gain1q (HR 3.22, 95% CI 1.89–5.49, *p* < 0.0001 (Fig. 2B), with no significant differences between Gain1q and Normal1q groups. 4-year OS rate was 53% (95% CI 41–69%) Amp1q, 78% (95% CI 71–86%) Gain1q and 91% (95% CI 87–95%) in Normal1q.

294 patients of the ITT 1q population were randomized to maintenance (R2): 26/52 (50%) with Amp1q, 100/129 (78%) with Gain1q and 168/219 (77%) with Normal 1q. Reasons for discontinuation before R2 are listed in Fig. 1; there was a higher rate of discontinuation due to PD in the Amp1q group (13/52, 25%) compared to Gain1q (14/129, 11%) and Normal 1q (18/219, 8%). Patients’ characteristics of ITT 1q patients randomized to maintenance are summarized in the Supplementary Appendix (Table S2).

The PFS from second randomization (R2) was significantly inferior in patients with Amp1q vs. Normal1q (HR 3.05, 95% CI 1.69–5.51, *p* = 0.0002) and Gain1q vs. Normal 1q (HR 1.81, 95% CI 1.14–2.86, *p* = 0.0116) while only a trend was found in Amp1q vs. Gain1q (HR 1.69, 95% CI 0.93–3.06, *p* = 0.0858) at the current follow-up (Fig. S2). 3-year PFS rate was 46% (95% CI 30–70%) vs. 65% (95% CI 56–75%) vs. 80% (95% CI 74–86%) in Amp1q vs. Gain1q vs. Normal 1q groups.

### Subgroup analyses

**Fig. 3:**
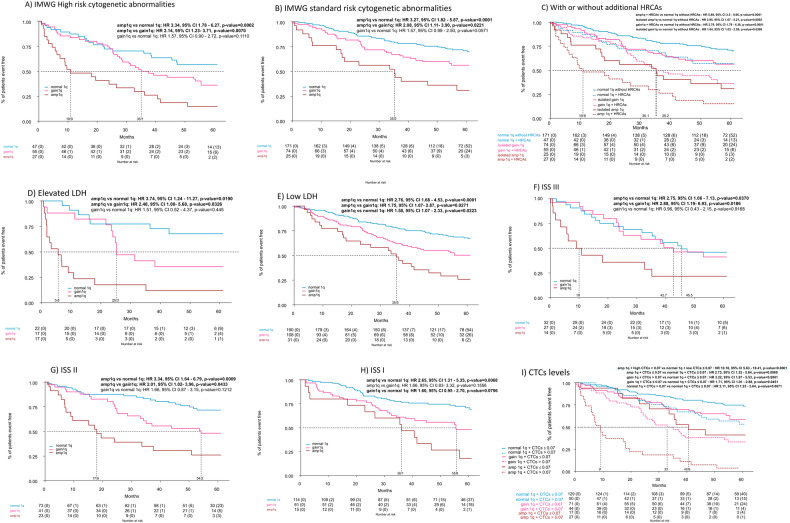
Progression-free survival in subgroups of interest. Progression-free survival from first randomization (R1) according to 1q alterations and concomitant cytogenetic risk (**A**–**C**), LDH levels (**D** and **E**), ISS (**F**–**H**), and circulating tumor cells (CTCs) levels (**I**).

Subgroup analyses for PFS confirmed the negative prognostic impact of Amp1q vs. Gain1q and Normal q1 in all subgroups of patients (Fig. S3). Of interest, the co-occurrence of Amp1q with other baseline risk features (Fig. 3) identifies a group of patients with an extremely poor outcome [median PFS of high-risk cytogenetics + Amp1q 10.9 months (95% CI 7.3–36.1); High LDH + Amp1q 5.6 months (95% CI 1.9–17.9); ISS3 + Amp1q 10 months (95% CI 5.8–NR)]. Interestingly, Amp1q without additional high-risk chromosomal abnormalities (HRCA) had a similar outcome compared with Gain1q with additional HRCA [Fig. 3C, median PFS 35.2 months (95% CI 17.1–NR) vs. 35.1 months (95% CI 28.4–NR); HR 1.30, 95% CI 0.54–3.14, *p* = 0.56]. However, the worst outcome was detected in Amp1q patients with additional HRCA, suggesting that there is an additive effect of additional HRCA even in the context of very high-risk abnormalities like Amp1q.

We further analyzed the relation of Gain1q and Amp1q with a novel prognostic marker described in the FORTE cohort, namely the levels of baseline circulating tumor cells (CTCs) in the peripheral blood with a cutoff of 0.07% by multiparametric flow cytometry [31]. As with other baseline risk features, the coexistence of Amp1q and high CTCs led to the worst PFS [median PFS 9 months (95% CI 6.3–18.1), Fig. 3I].

**Fig. 4:**
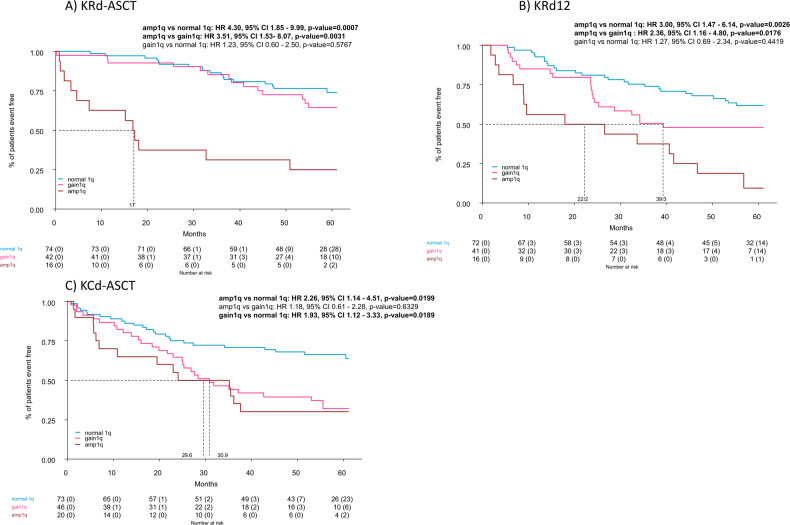
Progression-free survival from first randomization (R1) according to treatment received and 1q alterations. **A** KRd-ASCT, **B** KRd12, and **C** KCd-ASCT.

Regarding treatment, the negative impact of Amp1q was evident in all treatment arms (Figs. 4 and S3); for what concerns Gain1q, patients treated with KRd-ASCT arm showed a superimposable PFS compared to Normal1q [4 year PFS 72% (95% CI 60–88%) for Gain1q vs. 77% (95% CI 67–87%) for Normal1q; HR 1.23, 95% CI 0.60–2.50, *p* = 0.5777]. PFS of Gain1q vs Normal 1q was still inferior in patients treated with KCd-ASCT [4-y PFS: 39% (95% CI 27–57%) vs. 68% (95% CI 58–80%), HR 1.93, 95% CI 1.12–3.33, *p* = 0.0189], while in patients treated with KRd12 a non-significant trend was noted [4-y PFS: 48% (95% CI 34–67%) vs. 68% (95% CI 58–80%), HR 1.27, 95% CI 0.69–2.34, *p* = 0.4419].

Of note, the achievement of premaintenance MRD negativity by MFC 10^−5^ improved the outcome of all patients’ groups (Fig. S4B); nevertheless, Ampl1q still retained its prognostic significance (Fig. S4B). In patients achieving 1-year sustained MRD negativity, no significant PFS differences can be found according to 1q subgroups at current follow-up (Fig. S4C). On the other hand, Amp1q patients not achieving MRD negativity have a median PFS of only 7.3 months (95% CI 5.8–17.1), representing a very high-risk population (Fig. S4A).

### RNAseq of malignant plasma cells according to 1q copies

In order to compare groups with a similar sample size, in the ANOVA-like analysis of RNAseq data according to 1q copies [32], we randomly selected a population of patients with Normal 1q and Gain1q to match the size of the Amp1q group (analyzed patients are listed in Table S3).

Patients with a concomitant t(4;14) translocation were excluded from the analysis because the high expression of FGFR3 in these cases interacted with the clustering of 1q-defined subgroups. Indeed, in our case series, this effect was pronounced in Amp1q cases, where patients with the cooccurrence of t(4;14) clustered together (Fig. S5A).

**Fig. 5:**
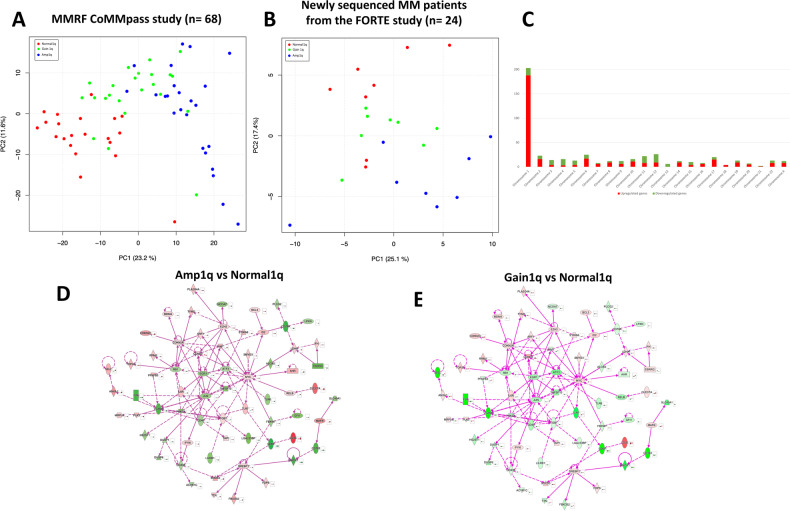
Transcriptomic deregulation according to 1q copies. Principal component analysis using RNAseq data was able to separate NDMM patients according to 1q copy numbers in the MMRF CoMMpass study (**A**). The separation was maintained when applied to a validation cohort of newly sequenced NDMM patients from the FORTE trial (**B**). Number of differentially expressed genes divided according to chromosome number are presented in Panel **C**. Ingenuity Pathway connectivity analysis of differentially expressed genes in Amp1q vs. Normal1q (**D**) and Gain1q vs. Normal1q (**E**) shows a deregulated gene network centered on Myc. Green color indicates down-regulation and red color up-regulation.

An ANOVA-like analysis of RNAseq data of 68 patients enrolled in the CoMMpass study (Table S3) showed 498 differentially expressed genes that were able to separate 1q-defined groups in a Principal Component Analysis (Fig. 5A). Clustering according to 1q groups is shown in Fig. S5B. The list of differentially expressed genes in Amp1q, Gain1q, and Normal1q is provided in Table S4. The identified genes were able to separate 1q-defined groups also in an independent population of 24 patients enrolled in the FORTE trial that were not included in the CoMMpass study (Fig. 5B).

Of note, many (203/498) but not all differentially expressed genes (DEGs) were located in chromosome 1 (Fig. 5C). Moreover, 188 of the 203 DEGs in chromosome 1 were upregulated consistently with the additional 1q copies.

An ingenuity pathways analysis showed that apoptosis signaling (*z*-score 2.646) and p38 MAPK signaling pathways (*z*-score 2.646) were the most deregulated pathways in Amp1q patients (Table S5).

Ingenuity pathway connectivity analysis of differentially expressed genes in Amp1q vs. Normal1q (Fig. 5D) and Gain1q vs. Normal1q (Fig. 5E) showed a deregulated gene network that was centered on the Myc gene.

Notably, genes that were deregulated in Gain1q cases, have usually a more pronounced deregulation in Amp1q cases (e.g. EGR1, Fig. S5C). However, some genes (e.g. AHR and RELB, Fig. S5C) showed a change in expression direction in Gain1q vs. Amp1q cases.

Of note, CD55, a gene located in chromosome 1q, which is known to promote resistance to complement-dependent cytotoxicity induced by anti-CD38 monoclonal antibodies (e.g. daratumumab) [33], was upregulated in Gain and especially Amp1q cases. (Fig. S5C).

Moreover, the GPRC5D gene, a relevant immunotherapeutic target in MM [34], despite being located outside of chromosome 1q, was upregulated in Gain and especially Amp1q patients in our analysis (Fig. S5C).

## Discussion

We demonstrated that the number of 1q copies detected by FISH is an independent prognostic marker in a population of transplant-eligible NDMM patients treated with carfilzomib-based combinations.

Amp1q (≥4 copies of 1q) was associated with a very poor PFS and OS compared with Normal but also with Gain1q, while patients with Gain1q (3 copies of 1q), showed an intermediate outcome.

The different prognostic impact of Gain1q and Amp1q is in line with some studies [9, 14, 16], while other reports showed similar OS [15, 21, 35].

Technical variability in calling 1q status, differences in follow-up time, differences in treatment strategies, and a different distribution of concomitant high-risk features may account for some of these discrepancies.

No consensus exists on the optimal cutoffs to define Gain1q vs. Amp1q. Here we used a 20% cut-off of nuclei with ≥4 copies of 1q to define Amp1q positivity, and an Epanechnikov kernel-smoothed estimated hazard rate showed that the risk of progression is higher if the clonal size of cells with ≥4 copies of 1q is higher.

While using a more stringent cut-off may identify a smaller subset of patients with a higher risk of progression, a recent report showed that even the presence of small 1q positive subclones identified through single-cell technology, may affect prognosis [36]. Indeed, the selection of 1q subclones emerging at relapse may show a similar negative impact on PFS and OS compared to patients who have the alteration from diagnosis [36].

Defining the prognostic impact of Amp 1q vs. Gain1q is meaningful if we can also identify subgroups of patients who can benefit from different treatment approaches. In a retrospective analysis of patients treated with bortezomib–lenalidomide–dexamethasone (VRd), Amp1q predicted a poor PFS regardless of other cytogenetic abnormalities, while patients with Gain1q had a dismal outcome only when Gain1q was associated with t(4;14), or t(14;16) or del (17p) [14].

In the E1A11 trial, standard risk NDMM patients not intended for upfront transplant were randomized to VRd vs. KRd induction followed by indefinite or 2-year R maintenance. Since the enrollment of t(4;14), del(1p), and Gain/Amp1q patients were allowed, a post-hoc analysis focusing on chromosome 1 alterations was performed [37]. Gain1q and Amp1q patients did show poor PFS with either VRd or KRd [37]. However, looking at OS, KRd seemed to overcome the negative impact of Gain1q, but not Amp1q.

In our study, both Gain1q and especially Amp1q showed a lower PFS compared to patients with Normal 1q, independently from the presence of other high-risk features. Nevertheless, treatment with KRd induction-ASCT-KRd consolidation seemed to overcome the negative prognostic value of Gain1q. Amp1q remained strongly associated with poor prognostic in all treatment arms, and many of these patients did not reach the maintenance phase due to early progression. Moreover, when Amp1q was associated with other high-risk features (e.g. other high-risk cytogenetic abnormalities, high LDH, ISS 3, high CTCs) or when patients did not reach bone marrow MRD negativity, PFS was <12 months, clearly identifying a patient population with a very high unmet medical need. Indeed, despite isolated Amp1q already predicted an adverse outcome, there is a clear additive effect of additional poor prognostic factors even in Amp1q-positive patients.

Achieving and sustaining over time MRD negativity was the only factor ameliorating the prognosis of Amp1q patients, indicating the need to explore novel treatment options to pursue sustained MRD in these patients, such as early intensification of treatment in patients harboring Amp1q not reaching an MRD negativity or not sustaining it over time.

We analyzed RNAseq to detect DEGs associated with Gain1q and Amp1q that can shed light on druggable targets/pathways to be further tested in this high-risk patient population.

The concept that copy number alterations can cause a functional impact on the transcriptome has been shown before [38]. In that study, 1q alterations were associated with the greatest impact on gene expression, deregulating pathways related to cell cycle, proliferation, and expression of immunotherapy targets [38].

In our study, we found that deregulation in apoptosis signaling and p38 MAPK signaling pathways and a gene network centered on Myc may contribute to the high-risk behavior associated with additional 1q copies.

Moreover, we found in Amp1q cases, an upregulation of CD55 that may reduce the efficacy of anti-CD38 monoclonal antibodies relying on complement-dependent cytotoxicity as the main mechanism of action. An upregulation of GPRC5D can be found in Amp1q cases as well in our cohort and bispecific antibodies [39]/CAR T cells [40] targeting GPRC5D are under evaluation in clinical trials.

Subgroup analyses on Amp1q patients treated with these agents are warranted to understand if the transcriptomic results translate into a different clinical benefit with these therapies.

The exclusion from the transcriptomic analysis of patients with concomitant t(4;14) may have reduced the risk of cluster bias. However, though we have not observed a clear effect of other concomitant alterations, the impact of the coexistence of other alterations cannot be completely ruled out. Indeed, the main limitation of our analysis is that we used bulk RNAseq, while single-cell RNAseq may identify more precisely the transcription alterations in 1q positive cells. In a single-cell RNA seq study addressing +1q cells, an upregulation of mitochondrial oxidative phosphorylation causing increased reactive oxygen species and reduced energy stress, compared with cells without +1q, was found [41]. Using single-cell RNAseq, the MYC gene expression signature is enriched amongst +1q samples, with MYC cooperating with MCL1 to enrich the MYC gene expression signature in +1q samples. This is indeed consistent with our analysis using bulk RNAseq data.

In conclusion, in our cohort, Amp1q identifies patients with very high-risk MM, while Gain1q patients are at intermediate risk of progression and/or death and may benefit from the KRd-ASCT-KRd approach. Additional copies of 1q induced relevant transcriptomic changes in MM cells, and it may be worth exploring the use of specific agents in this patient subset.

### Supplementary information

SUPPLEMENTARY APPENDIX

## Data Availability

After the publication of this article, data collected for this analysis and related documents will be made available to others upon reasonably justified request, which needs to be written and addressed to the attention of the corresponding author FG at the following e-mail address: francesca.gay@unito.it. The sponsor of the UNITO-MM-01/FORTE trial, the University of Torino (Italy), via the corresponding author FG, is responsible for evaluating and eventually accepting or refusing every request to disclose data and their related documents, in compliance with the ethical approval conditions, in compliance with applicable laws and regulations, and in conformance with the agreements in place with the involved subjects, the participating institutions, and all the other parties directly or indirectly involved in the participation, conduct, development, management and evaluation of this analysis.
