# Supplementary material for: Additional copies of 1q negatively impact the outcome of multiple myeloma patients and induce transcriptomic deregulation in malignant plasma cells
Source: Blood Cancer J. 2024 Jun 7;14(1):94. doi: 10.1038/s41408-024-01075-x (PMC11161499; doi:10.1038/s41408-024-01075-x)
Supplement: Supplementary file 1 — SUPPLEMENTARY APPENDIX [file 41408_2024_1075_MOESM1_ESM.docx]

**SUPPLEMENTARY APPENDIX**

Supplementary methods

Sustained MRD negativity evaluation

1-year sustained MRD negativity was defined as two consecutive negative results, the first at premaintenance and the second at least 12 months apart. Patients who tested negative at premaintenance and had a subsequent positive MRD test result or relapsed within 12 months were considered positive for unsustained MRD. According to the ITT principle, patients who were positive before maintenance treatment, or missed the follow-up assessment of minimal residual disease, or had a positive MRD test at 1 year were considered positive for MRD.

**RNA sequencing data generation and analysis**

RNAseq data in the newly sequenced patients within the FORTE trial were generated using CD138+ selected BMPCs. Total RNA was isolated from samples using TriZol reagent (Thermo-Fisher Scientific) and following manufacturer’s instructions. Genomic DNA contaminations were removed from RNA samples with the Ambion® DNA-free kit (Thermo-Fisher Scientific). DNA-free RNA concentration and quality were estimated with NanoVuePlus Spectrophotometer (GE Healthcare, Little Chalfont, UK) and Agilent 2100 Bioanalyzer (Agilent Technologies, Santa Clara, CA, USA), respectively. Libraries for RNA sequencing were generated using TruSeq RNA Access sample preparation kit v2 (Illumina Inc., San Diego, CA, USA) following manufacturer’s instructions, using 100 ng of total RNA as input material.

Libraries were sequenced with NextSeq 500 sequencer (Illumina Inc.) generating 75-bp paired-end sequences.

The sequence analysis was performed removing adapters with skewer(1), mapping reads on hg38 genome with STAR(2), assigning gene level expression by RSEM(3) and annotating the ENSEMBL IDs with refGenome R package. Counts, log_2_FPKM and log_2_TPM matrices were generated using docker4seq sample2experiment function. The above-mentioned analysis was performed within the docker4seq framework(4). Differential expression analysis was done using the edgeR ANOVA-like procedure(5) (False Discovery Rate ≤ 0.1; absolute log2 Fold Change ≥ 1). Differentially expressed genes were analyzed using hierarchical clustering (Morpheus tool, data were Z-scored before clustering. Clustering was calculated using Euclidean distance and average linkage). Code is available upon request.

**Data deposition**

The access to the Interim Analysis 14 (IA 14) release of CoMMpass was approved by the Data. Access Use Committee and downloaded from https://research.themmrf.org/rp/download. CoMMpass data are deposited in the database of Genotypes and Phenotypes (dbGaP; StudyAccession phs000748.v7.p4 - https://www.ncbi.nlm.nih.gov/projects/gap/cgi-bin/study.cgi?study_id=phs000748.v7.p4).

Data used for the validation of differentially expressed genes in the FORTE cohort have been deposited in NCBI's Gene Expression Omnibus(6) (and are accessible through GEO Series accession number GSE193427).

**PFS analysis using 20% cut-off to define Gain 1q**

Using a 20% cut-off to define Gain 1q, 238/400 (59.5%) patients were classified as Normal 1q, 110/400 (27.5%) patients were classified as Gain 1q and 52/400 (13%) were classified as Amp 1q.

PFS from R1 was significantly inferior in the presence of Amp1q vs Normal1q (HR 3.19, 95% CI 2.15-4.72, p<0.0001) and Amp1q vs Gain1q (HR 2.01, 95% CI 1.34-3.03, P=0.0008). Patients with Gain1q had also a significantly shorter PFS compared with Normal 1q (HR 1.58, 95% CI 1.12-2.25 P=0.0010). 4-year PFS rate was 27% (95% CI 17%-42%) in Amp1q, 52% (95% CI 43%-63%) in Gain1q and 70% (95% CI 64%-76%) in Normal 1q.

Table S1. Response to treatment (ITT-analysis) according to 1q21 status.

|  | **Amp1q** | **Gain1q** | **Normal 1q** | **p-value** |
| --- | --- | --- | --- | --- |
|  | N=52 | N=129 | N=219 |  |
| **≥VGPR (premaintenance)** | 40 [77 (95% CI 63 – 87)] | 108 [84(95% CI 76-90)] | 183 [84(95% CI 78 - 88)] | 0.49 |
| **≥CR (premaintenance)** | 24 [46(95% CI 32 - 61)] | 60 [47(95% CI 38 - 55)] | 113 [52(95% CI 45 -58)] | 0.59 |
| **10^-5^ MRD neg by MFC**  **(premaintenance )** | 23 [44(95% CI 30 – 59]) | 71 [55(95% CI 46 - 64]) | 121 [55(95% CI 48 - 62)] | 0.35 |
| **1-year sustained 10-5 MRD**  **neg by MFC** | 10 [19(95% CI 10 - 33)] | 41 [32(95% CI 24 - 41)] | 81 [37(95% CI 31 - 44)] | 0.05 |

Percentage may not total 100 because of rounding. % calculated on the available patients.

**Abbreviations**: VGPR, very good partial response; CI, confidence interval; CR, complete response; MRD, minimal residual disease; neg, negativity; MFC, multiparameter flow cytometry; ITT, intention to treat.

Table S2. Patient characteristics of patients randomized for maintenance therapy (R2) FORTE trial according to 1q21 status.

|  |  | **FORTE population** | **1q Amp/Gain Not evaluable** | **Amp1q** | **Gain1q** | **Normal 1q** | **p-value*** |
| --- | --- | --- | --- | --- | --- | --- | --- |
|  |  | N=356 | N= 62 | N=26 | N=100 | N=168 |  |
| **Age** | Median (IQR) | 57- (52-62) | 55- (48-61) | 55 – (53-61) | 58 – (54-62) | 56- (52-61) | 0.41 |
| **ISS** | I | 194 (54) | 46 (74) | 10 (38) | 46 (46) | 92 (55) | 0.41 |
|  | II | 113 (32) | 10 (16) | 13 (50) | 35 (35) | 55 (32) |  |
|  | III | 49 (14) | 6 (10) | 3 (12) | 19 (19) | 32 (12) |  |
| **Cytogenetic abnormalities** | Standard Risk | 217 (72) | 5 (71) | 18 (69) | 59 (59) | 135 (81) | 0.38 |
|  | High Risk** | 83 (28) | 2 (29) | 8 (31) | 41 (41) | 32 (19) |  |
|  | Missing | 56 | 55 | 0 | 0 | 1 |  |
|  | del(17p) | 36 (12) | 2 (29) | 3 (12) | 11 (11) | 20 (12) | 1 |
|  | t(4;14) | 36 (12) | 0 | 3 (12) | 22 (22) | 11 (7) | 0.28 |
|  | t(14;16) | 16 (5) | 0 | 4 (15) | 10 (10) | 2 (1) | 0.49 |
| **LDH** | Normal | 308 (90) | 58 (95) | 19 (83) | 82 (85) | 149 (91) | 0.75 |
|  | High | 35 (10) | 3 (5) | 4 (17) | 14 (15) | 14 (9) |  |
|  | Missing | 13 | 1 | 3 | 4 | 5 |  |
| **Maintenance Therapy (R2)** | KR | 178 (50) | 38 (59) | 15 (58) | 46 (46) | 80 (48) | 0.38 |
|  | R | 178 (50) | 26 (41) | 11 (42) | 54 (54) | 88 (52) |  |

Percentage may not total 100 because of rounding. % calculated on the available patients. *Amp1q vs. Gain1q. **High risk-cytogenetic abnormalities: defined as del(17p), t(4;14) and/or t(14;16);

**Abbreviations**: IQR: interquartile range; ISS: International Staging System; LDH: lactate dehydrogenase; FISH: fluorescence in situ hybridization; KR, carfilzomib lenalidomide; R, lenalidomide

Table S3. List of CoMMpass patients analyzed according to 1q copies and presence/absence of concomitant chromosomal abnormalities.

| Patient ID | 1q group | t(14;16) Yes/No | del(17p) Yes/No | t(4;14) Yes/No | t(11;14) Yes/No | t(14;20) Yes/No | Del(13q)  Yes/No |
| --- | --- | --- | --- | --- | --- | --- | --- |
| MMRF1157 | Normal 1q | No | No | No | Yes | No | No |
| MMRF1092 | Normal 1q | No | No | No | No | No | No |
| MMRF1311 | Normal 1q | No | No | No | Yes | No | Yes |
| MMRF1309 | Normal 1q | No | No | No | No | No | No |
| MMRF1164 | Normal 1q | Yes | No | No | No | No | Yes |
| MMRF1068 | Normal 1q | No | No | No | No | No | No |
| MMRF1231 | Normal 1q | No | No | No | Yes | No | No |
| MMRF1037 | Normal 1q | No | No | No | No | No | No |
| MMRF1098 | Normal 1q | No | No | No | Yes | No | Yes |
| MMRF1331 | Normal 1q | No | No | No | **No** | No | Yes |
| MMRF1030 | Normal 1q | No | No | No | No | No | No |
| MMRF1108 | Normal 1q | No | No | No | No | No | No |
| MMRF1032 | Normal 1q | No | No | No | No | No | No |
| MMRF1348 | Normal 1q | No | No | No | No | No | Yes |
| MMRF1185 | Normal 1q | No | No | No | No | No | Yes |
| MMRF1151 | Normal 1q | No | No | No | No | No | No |
| MMRF1029 | Normal 1q | No | No | No | No | No | Yes |
| MMRF1270 | Normal 1q | No | No | No | No | No | Yes |
| MMRF1073 | Normal 1q | No | No | No | No | No | No |
| MMRF1352 | Normal 1q | No | No | No | Yes | No | No |
| MMRF1100 | Normal 1q | No | No | No | Yes | No | Yes |
| MMRF1413 | Gain 1q | No | No | No | No | No | No |
| MMRF1510 | Gain 1q | No | No | No | No | No | No |
| MMRF1274 | Gain 1q | No | No | No | No | No | Yes |
| MMRF1445 | Gain 1q | No | No | No | No | No | Yes |
| MMRF1523 | Gain 1q | No | No | No | Yes | No | No |
| MMRF1269 | Gain 1q | No | No | No | Yes | No | No |
| MMRF1082 | Gain 1q | No | No | No | Yes | No | No |
| MMRF1344 | Gain 1q | No | No | No | No | No | Yes |
| MMRF1454 | Gain 1q | Yes | No | No | No | No | Yes |
| MMRF1148 | Gain 1q | No | No | No | No | No | No |
| MMRF1447 | Gain 1q | No | No | No | No | No | No |
| MMRF1519 | Gain 1q | No | No | No | No | No | Yes |
| MMRF1045 | Gain 1q | No | No | No | No | No | No |
| MMRF1461 | Gain 1q | No | No | No | No | Yes | Yes |
| MMRF1285 | Gain 1q | No | No | No | No | No | Yes |
| MMRF1153 | Gain 1q | No | No | No | No | No | Yes |
| MMRF1189 | Gain 1q | No | No | No | Yes | No | No |
| MMRF1195 | Gain 1q | No | No | No | No | No | Yes |
| MMRF1167 | Gain 1q | No | No | No | No | No | Yes |
| MMRF1031 | Gain 1q | No | No | No | No | No | Yes |
| MMRF1129 | Gain 1q | No | No | No | No | No | Yes |
| MMRF1401 | Gain 1q | No | No | No | Yes | No | No |
| MMRF1502 | Gain 1q | No | No | No | No | No | Yes |
| MMRF2587 | Amp1q | No | No | No | No | No | Yes |
| MMRF1771 | Amp1q | No | No | No | No | No | No |
| MMRF1323 | Amp1q | No | No | No | No | No | Yes |
| MMRF2770 | Amp1q | No | No | No | No | No | Yes |
| MMRF1388 | Amp1q | No | No | No | No | No | No |
| MMRF2293 | Amp1q | No | No | No | No | No | Yes |
| MMRF1180 | Amp1q | Yes | No | No | No | No | Yes |
| MMRF2290 | Amp1q | No | No | No | No | No | Yes |
| MMRF1965 | Amp1q | Yes | No | No | No | No | Yes |
| MMRF1656 | Amp1q | No | No | No | No | No | No |
| MMRF1855 | Amp1q | Yes | No | No | No | No | Yes |
| MMRF2170 | Amp1q | No | No | No | No | No | Yes |
| MMRF2231 | Amp1q | No | No | No | No | No | Yes |
| MMRF2762 | Amp1q | Yes | No | No | No | No | Yes |
| MMRF1107 | Amp1q | No | No | No | No | No | Yes |
| MMRF1715 | Amp1q | No | No | No | No | Yes | Yes |
| MMRF2473 | Amp1q | No | No | No | No | No | Yes |
| MMRF1886 | Amp1q | No | No | No | Yes | No | No |
| MMRF2549 | Amp1q | No | No | No | No | No | Yes |
| MMRF2106 | Amp1q | Yes | No | No | No | No | Yes |
| MMRF1079 | Amp1q | No | No | No | No | No | Yes |
| MMRF2455 | Amp1q | No | No | No | No | Yes | Yes |
| MMRF2732 | Amp1q | No | No | No | No | No | No |
| MMRF1780 | Amp1q | No | No | No | Yes | No | No |

Table S4. List of differentially expressed genes by ANOVA-like analysis in Amp1q and Gain1q patients vs Normal1q patients.

| Gene | Gain1q vs Normal1q | Amp1q vs Normal1q | Log Counts Per Milion | F | P Value | False Discovery Rate |
| --- | --- | --- | --- | --- | --- | --- |
| SFRP2:ENSG00000145423_chr4:153780591-153789083_protein_coding | 11.36 | 8.75 | 5.63 | 16.02 | 1.87E-06 | 0.00 |
| MAFA:ENSG00000182759_chr8:143419182-143430732_protein_coding | 0.75 | 8.04 | 4.33 | 29.95 | 4.00E-10 | 0.00 |
| THY1:ENSG00000154096_chr11:119415476-119424985_protein_coding | 10.24 | 6.89 | 5.47 | 16.84 | 1.07E-06 | 0.00 |
| MYO1E:ENSG00000157483_chr15:59132434-59372871_protein_coding | 4.78 | 6.84 | 4.93 | 20.38 | 1.06E-07 | 0.00 |
| LINC01287:ENSG00000234722_chr7:153355365-153413985_lncRNA | 3.68 | 5.59 | 4.47 | 20.20 | 1.19E-07 | 0.00 |
| CD3E:ENSG00000198851_chr11:118304730-118316175_protein_coding | 4.51 | 5.40 | 4.30 | 10.45 | 0.00010657 | 0.00 |
| WSCD1:ENSG00000179314_chr17:6057807-6124427_protein_coding | 6.00 | 5.18 | 4.73 | 12.27 | 2.70E-05 | 0.00 |
| BCHE:ENSG00000114200_chr3:165772904-165837462_protein_coding | 5.79 | 4.97 | 5.53 | 13.15 | 1.42E-05 | 0.00 |
| CDHR1:ENSG00000148600_chr10:84194537-84219621_protein_coding | 0.76 | 4.96 | 4.74 | 10.52 | 0.00010118 | 0.00 |
| MAGEC2:ENSG00000046774_chrX:142202342-142205290_protein_coding | 3.93 | 4.36 | 4.44 | 14.75 | 4.53E-06 | 0.00 |
| CLEC7A:ENSG00000172243_chr12:10116777-10130258_protein_coding | 0.91 | 4.05 | 4.63 | 11.58 | 4.53E-05 | 0.00 |
| MYBPC2:ENSG00000086967_chr19:50432892-50466321_protein_coding | 5.21 | 4.01 | 5.28 | 8.22 | 0.00062095 | 0.01 |
| NES:ENSG00000132688_chr1:156668763-156677407_protein_coding | 3.02 | 3.85 | 7.72 | 7.71 | 0.00094515 | 0.01 |
| NUAK1:ENSG00000074590_chr12:106063340-106140033_protein_coding | 1.53 | 3.67 | 4.69 | 4.31 | 0.01713054 | 0.09 |
| UCHL1:ENSG00000154277_chr4:41256413-41268455_protein_coding | 3.92 | 3.38 | 5.63 | 6.49 | 0.00260388 | 0.02 |
| MTCO1P12:ENSG00000237973_chr1:631074-632616_unprocessed_pseudogene | 1.88 | 3.27 | 5.36 | 9.01 | 0.00033003 | 0.01 |
| S100A4:ENSG00000196154_chr1:153543613-153550136_protein_coding | 1.39 | 3.26 | 6.42 | 12.76 | 1.89E-05 | 0.00 |
| CRIP1:ENSG00000213145_chr14:105486317-105488947_protein_coding | 1.20 | 3.23 | 5.59 | 14.00 | 7.69E-06 | 0.00 |
| DCC:ENSG00000187323_chr18:52340197-53535903_protein_coding | 3.43 | 3.13 | 4.08 | 9.28 | 0.00026656 | 0.00 |
| JUP:ENSG00000173801_chr17:41754604-41786931_protein_coding | 2.32 | 3.10 | 5.18 | 8.66 | 0.00043555 | 0.01 |
| CD109:ENSG00000156535_chr6:73695785-73828316_protein_coding | -0.28 | 3.00 | 5.33 | 8.62 | 0.00045069 | 0.01 |
| COL18A1:ENSG00000182871_chr21:45405165-45513720_protein_coding | -1.74 | 2.97 | 4.11 | 16.81 | 1.09E-06 | 0.00 |
| FCRL3:ENSG00000160856_chr1:157674321-157700769_protein_coding | 1.15 | 2.94 | 6.05 | 8.53 | 0.00048355 | 0.01 |
| LMNA:ENSG00000160789_chr1:156082573-156140089_protein_coding | 0.56 | 2.93 | 9.62 | 17.01 | 9.53E-07 | 0.00 |
| RND3:ENSG00000115963_chr2:150468195-150539011_protein_coding | 1.71 | 2.91 | 6.71 | 5.67 | 0.00522521 | 0.04 |
| H1-0:ENSG00000189060_chr22:37805093-37807436_protein_coding | 1.38 | 2.86 | 6.17 | 11.34 | 5.43E-05 | 0.00 |
| ANXA1:ENSG00000135046_chr9:73151865-73170393_protein_coding | 0.75 | 2.76 | 6.07 | 7.83 | 0.0008566 | 0.01 |
| MAFB:ENSG00000204103_chr20:40685848-40689236_protein_coding | 1.03 | 2.75 | 4.78 | 4.80 | 0.01115212 | 0.07 |
| AC139491.2:ENSG00000248596_chr5:176143085-176185155_lncRNA | -1.71 | 2.73 | 4.38 | 6.15 | 0.00346142 | 0.03 |
| CAPN5:ENSG00000149260_chr11:77066961-77126155_protein_coding | 2.39 | 2.64 | 4.12 | 6.31 | 0.00303627 | 0.03 |
| TAGLN2:ENSG00000158710_chr1:159918107-159925507_protein_coding | 0.31 | 2.63 | 7.58 | 34.52 | 3.72E-11 | 0.00 |
| GNAL:ENSG00000141404_chr18:11689264-11885685_protein_coding | 1.13 | 2.57 | 4.33 | 8.47 | 0.00050647 | 0.01 |
| GREB1:ENSG00000196208_chr2:11482341-11642788_protein_coding | 1.56 | 2.57 | 4.46 | 4.52 | 0.01430124 | 0.08 |
| CDKN2C:ENSG00000123080_chr1:50960745-50974634_protein_coding | 1.52 | 2.53 | 4.32 | 12.31 | 2.62E-05 | 0.00 |
| PKP2:ENSG00000057294_chr12:32790755-32896777_protein_coding | 1.08 | 2.52 | 5.17 | 5.50 | 0.00606425 | 0.04 |
| SOCS3:ENSG00000184557_chr17:78356778-78360077_protein_coding | 1.55 | 2.50 | 7.51 | 4.47 | 0.01494265 | 0.08 |
| KIF21B:ENSG00000116852_chr1:200969390-201023700_protein_coding | 1.66 | 2.49 | 6.65 | 9.11 | 0.00030548 | 0.01 |
| S100A10:ENSG00000197747_chr1:151982915-151993859_protein_coding | 0.22 | 2.49 | 6.02 | 18.95 | 2.65E-07 | 0.00 |
| PTP4A3:ENSG00000184489_chr8:141391995-141432454_protein_coding | 2.48 | 2.48 | 8.39 | 8.02 | 0.00072924 | 0.01 |
| CENPF:ENSG00000117724_chr1:214603195-214664571_protein_coding | 1.59 | 2.42 | 4.56 | 19.40 | 1.98E-07 | 0.00 |
| DEPP1:ENSG00000165507_chr10:44970981-44978809_protein_coding | 0.25 | 2.34 | 6.77 | 6.41 | 0.00277959 | 0.03 |
| ITGB7:ENSG00000139626_chr12:53191323-53207282_protein_coding | 0.08 | 2.33 | 10.59 | 7.48 | 0.00113812 | 0.01 |
| CFB:ENSG00000243649_chr6:31945650-31952084_protein_coding | 4.50 | 2.23 | 4.47 | 18.00 | 4.92E-07 | 0.00 |
| ASPM:ENSG00000066279_chr1:197084127-197146694_protein_coding | 1.41 | 2.18 | 4.76 | 19.09 | 2.42E-07 | 0.00 |
| FBXO32:ENSG00000156804_chr8:123497889-123541206_protein_coding | -0.73 | 2.18 | 6.41 | 20.60 | 9.23E-08 | 0.00 |
| AL133467.1:ENSG00000258572_chr14:95516136-95517911_lncRNA | 2.59 | 2.17 | 4.79 | 10.02 | 0.00014912 | 0.00 |
| STEAP3:ENSG00000115107_chr2:119223831-119265652_protein_coding | 1.68 | 2.15 | 4.24 | 10.64 | 9.27E-05 | 0.00 |
| AHR:ENSG00000106546_chr7:16916359-17346152_protein_coding | -0.26 | 2.12 | 4.06 | 7.16 | 0.00147978 | 0.02 |
| PBXIP1:ENSG00000163346_chr1:154944076-154956123_protein_coding | 0.21 | 2.11 | 8.78 | 37.86 | 7.19E-12 | 0.00 |
| TSPAN7:ENSG00000156298_chrX:38561370-38688920_protein_coding | 2.17 | 2.10 | 5.46 | 5.67 | 0.00523538 | 0.04 |
| SELL:ENSG00000188404_chr1:169690665-169711702_protein_coding | -2.95 | 2.09 | 7.05 | 14.65 | 4.85E-06 | 0.00 |
| DUXAP9:ENSG00000225210_chr14:19062316-19131167_transcribed_processed_pseudogene | 1.82 | 1.94 | 5.58 | 5.81 | 0.00461641 | 0.04 |
| AL589743.1:ENSG00000244306_chr14:19268853-19337730_transcribed_processed_pseudogene | 1.64 | 1.92 | 4.47 | 9.86 | 0.00016875 | 0.00 |
| C1orf21:ENSG00000116667_chr1:184387029-184629019_protein_coding | 1.15 | 1.92 | 5.00 | 5.40 | 0.0066076 | 0.05 |
| PLEKHA7:ENSG00000166689_chr11:16777297-17014414_protein_coding | 1.05 | 1.89 | 5.04 | 4.66 | 0.01258965 | 0.07 |
| PFKFB3:ENSG00000170525_chr10:6144934-6254644_protein_coding | -0.40 | 1.89 | 5.72 | 10.37 | 0.00011372 | 0.00 |
| TOP2A:ENSG00000131747_chr17:40388525-40417896_protein_coding | 1.48 | 1.89 | 4.66 | 10.73 | 8.60E-05 | 0.00 |
| RGL1:ENSG00000143344_chr1:183636085-183928531_protein_coding | -0.97 | 1.88 | 4.58 | 7.73 | 0.0009261 | 0.01 |
| E2F2:ENSG00000007968_chr1:23506438-23531233_protein_coding | 1.91 | 1.88 | 5.00 | 11.65 | 4.30E-05 | 0.00 |
| FAM189B:ENSG00000160767_chr1:155247205-155255483_protein_coding | 0.91 | 1.86 | 4.35 | 52.81 | 1.05E-14 | 0.00 |
| PIM1:ENSG00000137193_chr6:37170152-37175428_protein_coding | -0.17 | 1.86 | 8.07 | 13.18 | 1.38E-05 | 0.00 |
| PLTP:ENSG00000100979_chr20:45898621-45912155_protein_coding | -0.34 | 1.84 | 5.80 | 6.35 | 0.00292776 | 0.03 |
| HDGF:ENSG00000143321_chr1:156742109-156766925_protein_coding | 1.05 | 1.82 | 7.79 | 92.06 | 2.55E-20 | 0.00 |
| ADAM15:ENSG00000143537_chr1:155050566-155062775_protein_coding | 0.87 | 1.82 | 6.03 | 30.17 | 3.56E-10 | 0.00 |
| FCMR:ENSG00000162894_chr1:206903317-206923247_protein_coding | -1.24 | 1.80 | 4.13 | 12.31 | 2.63E-05 | 0.00 |
| RUSC1:ENSG00000160753_chr1:155320894-155331114_protein_coding | 0.96 | 1.80 | 4.25 | 51.32 | 1.91E-14 | 0.00 |
| H3P6:ENSG00000235655_chr2:174719908-174720318_processed_pseudogene | 0.71 | 1.79 | 8.02 | 30.40 | 3.15E-10 | 0.00 |
| TRIM47:ENSG00000132481_chr17:75874164-75878581_protein_coding | 0.26 | 1.79 | 4.74 | 13.65 | 9.89E-06 | 0.00 |
| PLK3:ENSG00000173846_chr1:44800377-44805990_protein_coding | 0.28 | 1.78 | 4.91 | 9.72 | 0.00018847 | 0.00 |
| TLR7:ENSG00000196664_chrX:12867072-12890361_protein_coding | -1.90 | 1.75 | 4.09 | 7.94 | 0.00078177 | 0.01 |
| TP53BP2:ENSG00000143514_chr1:223779893-223845954_protein_coding | 0.37 | 1.74 | 5.79 | 26.04 | 3.52E-09 | 0.00 |
| TYMS:ENSG00000176890_chr18:657653-673578_protein_coding | 1.29 | 1.74 | 4.82 | 14.17 | 6.82E-06 | 0.00 |
| S100A11:ENSG00000163191_chr1:152032506-152047907_protein_coding | 0.40 | 1.74 | 6.14 | 13.56 | 1.05E-05 | 0.00 |
| SDE2:ENSG00000143751_chr1:225982702-225999343_protein_coding | 0.46 | 1.72 | 6.18 | 27.70 | 1.38E-09 | 0.00 |
| RAB29:ENSG00000117280_chr1:205767986-205775482_protein_coding | 0.73 | 1.71 | 5.27 | 8.66 | 0.0004367 | 0.01 |
| S100A6:ENSG00000197956_chr1:153534599-153536244_protein_coding | 0.23 | 1.71 | 7.36 | 9.41 | 0.00023948 | 0.00 |
| CHML:ENSG00000203668_chr1:241628853-241640254_protein_coding | 0.98 | 1.71 | 6.20 | 21.93 | 4.04E-08 | 0.00 |
| SGK1:ENSG00000118515_chr6:134169246-134318112_protein_coding | -0.04 | 1.71 | 7.34 | 9.35 | 0.00025188 | 0.00 |
| PERP:ENSG00000112378_chr6:138088505-138107419_protein_coding | 0.62 | 1.70 | 6.99 | 7.25 | 0.00137404 | 0.02 |
| PIM3:ENSG00000198355_chr22:49960768-49964072_protein_coding | 0.16 | 1.70 | 6.39 | 14.88 | 4.11E-06 | 0.00 |
| TMCC2:ENSG00000133069_chr1:205227946-205285632_protein_coding | -0.07 | 1.70 | 4.27 | 6.81 | 0.00199218 | 0.02 |
| CKS1B:ENSG00000173207_chr1:154974653-154979251_protein_coding | 0.92 | 1.69 | 4.86 | 18.06 | 4.74E-07 | 0.00 |
| THBS3:ENSG00000169231_chr1:155195588-155209051_protein_coding | 0.83 | 1.69 | 4.65 | 34.51 | 3.73E-11 | 0.00 |
| MKI67:ENSG00000148773_chr10:128096659-128126423_protein_coding | 1.39 | 1.67 | 5.34 | 10.79 | 8.22E-05 | 0.00 |
| INAVA:ENSG00000163362_chr1:200891048-200915742_protein_coding | 0.22 | 1.67 | 4.31 | 4.32 | 0.01698979 | 0.09 |
| TPM3:ENSG00000143549_chr1:154155304-154194648_protein_coding | 0.49 | 1.64 | 8.18 | 47.42 | 9.64E-14 | 0.00 |
| BACH2:ENSG00000112182_chr6:89926528-90296908_protein_coding | 0.27 | 1.64 | 4.04 | 8.87 | 0.00036765 | 0.01 |
| CAPN2:ENSG00000162909_chr1:223701593-223776018_protein_coding | 1.00 | 1.63 | 8.71 | 20.01 | 1.34E-07 | 0.00 |
| ID2:ENSG00000115738_chr2:8678845-8684461_protein_coding | 1.25 | 1.62 | 5.98 | 7.36 | 0.0012572 | 0.01 |
| DIRAS1:ENSG00000176490_chr19:2714567-2721372_protein_coding | -0.30 | 1.60 | 4.18 | 6.27 | 0.00311748 | 0.03 |
| LGALSL:ENSG00000119862_chr2:64453969-64461381_protein_coding | 0.57 | 1.60 | 5.13 | 11.33 | 5.46E-05 | 0.00 |
| PLA2G4A:ENSG00000116711_chr1:186828949-186988981_protein_coding | 1.10 | 1.60 | 4.34 | 13.66 | 9.81E-06 | 0.00 |
| TTN:ENSG00000155657_chr2:178525989-178830802_protein_coding | -0.76 | 1.59 | 6.28 | 6.94 | 0.00177469 | 0.02 |
| MSTO2P:ENSG00000203761_chr1:155745829-155750137_unprocessed_pseudogene | 1.00 | 1.59 | 5.13 | 21.77 | 4.46E-08 | 0.00 |
| IL6R:ENSG00000160712_chr1:154405193-154469450_protein_coding | 1.14 | 1.59 | 8.60 | 11.01 | 6.97E-05 | 0.00 |
| ZBTB37:ENSG00000185278_chr1:173868082-173903549_protein_coding | 0.81 | 1.57 | 4.66 | 16.40 | 1.45E-06 | 0.00 |
| SHC1:ENSG00000160691_chr1:154962298-154974395_protein_coding | 0.88 | 1.56 | 8.13 | 23.92 | 1.21E-08 | 0.00 |
| CBX6:ENSG00000183741_chr22:38861422-38872249_protein_coding | 0.18 | 1.56 | 6.67 | 20.49 | 9.89E-08 | 0.00 |
| TOR1AIP2:ENSG00000169905_chr1:179839967-179877803_protein_coding | 0.62 | 1.56 | 6.60 | 42.49 | 8.33E-13 | 0.00 |
| TUBA1A:ENSG00000167552_chr12:49184795-49189080_protein_coding | -1.66 | 1.56 | 4.24 | 13.94 | 8.00E-06 | 0.00 |
| ARPC5:ENSG00000162704_chr1:183620846-183635783_protein_coding | 0.67 | 1.56 | 6.85 | 36.44 | 1.43E-11 | 0.00 |
| LAMTOR2:ENSG00000116586_chr1:156054782-156058506_protein_coding | 0.75 | 1.54 | 5.60 | 57.75 | 1.55E-15 | 0.00 |
| OPN3:ENSG00000054277_chr1:241590102-241677376_protein_coding | 0.87 | 1.54 | 4.70 | 17.78 | 5.72E-07 | 0.00 |
| HOMER2:ENSG00000103942_chr15:82836946-82986153_protein_coding | 0.43 | 1.53 | 5.17 | 5.40 | 0.00656831 | 0.05 |
| FKBP5:ENSG00000096060_chr6:35573585-35728583_protein_coding | 1.30 | 1.52 | 7.69 | 5.61 | 0.0054798 | 0.04 |
| RRM2:ENSG00000171848_chr2:10120698-10211725_protein_coding | 1.57 | 1.52 | 5.45 | 8.70 | 0.00042125 | 0.01 |
| SMOX:ENSG00000088826_chr20:4120980-4187747_protein_coding | 0.63 | 1.51 | 5.44 | 4.64 | 0.01284427 | 0.08 |
| AL365181.3:ENSG00000272405_chr1:156641666-156644887_lncRNA | 1.32 | 1.49 | 5.33 | 4.45 | 0.01513687 | 0.09 |
| CCT3:ENSG00000163468_chr1:156308968-156367873_protein_coding | 0.88 | 1.49 | 8.08 | 50.94 | 2.22E-14 | 0.00 |
| CHD1L:ENSG00000131778_chr1:147242654-147295765_protein_coding | 0.85 | 1.49 | 5.61 | 37.11 | 1.03E-11 | 0.00 |
| SFT2D2:ENSG00000213064_chr1:168225938-168253021_protein_coding | 0.23 | 1.48 | 6.41 | 40.79 | 1.81E-12 | 0.00 |
| PELI1:ENSG00000197329_chr2:64092652-64144420_protein_coding | 0.06 | 1.48 | 9.46 | 8.92 | 0.0003532 | 0.01 |
| EMP3:ENSG00000142227_chr19:48321509-48330553_protein_coding | 0.46 | 1.47 | 6.85 | 8.81 | 0.00038709 | 0.01 |
| GLMP:ENSG00000198715_chr1:156290089-156295689_protein_coding | 0.93 | 1.47 | 5.76 | 33.23 | 7.15E-11 | 0.00 |
| TAF5L:ENSG00000135801_chr1:229593111-229626047_protein_coding | 0.51 | 1.47 | 5.34 | 34.24 | 4.29E-11 | 0.00 |
| PMVK:ENSG00000163344_chr1:154924740-154936719_protein_coding | 0.51 | 1.47 | 6.36 | 33.55 | 6.08E-11 | 0.00 |
| CD55:ENSG00000196352_chr1:207321532-207386804_protein_coding | 0.18 | 1.46 | 7.28 | 14.61 | 4.99E-06 | 0.00 |
| TOR3A:ENSG00000186283_chr1:179082070-179098023_protein_coding | 0.86 | 1.46 | 8.56 | 23.80 | 1.31E-08 | 0.00 |
| H2AJ:ENSG00000246705_chr12:14774383-14778002_protein_coding | -0.30 | 1.46 | 5.42 | 12.13 | 3.00E-05 | 0.00 |
| AGO2:ENSG00000123908_chr8:140520156-140635633_protein_coding | 0.13 | 1.45 | 4.92 | 17.52 | 6.80E-07 | 0.00 |
| H3-3A:ENSG00000163041_chr1:226061851-226073212_protein_coding | 0.51 | 1.45 | 8.29 | 30.79 | 2.56E-10 | 0.00 |
| EZR:ENSG00000092820_chr6:158765741-158819368_protein_coding | 0.05 | 1.45 | 9.08 | 10.87 | 7.73E-05 | 0.00 |
| IL27RA:ENSG00000104998_chr19:14031762-14053218_protein_coding | 0.10 | 1.44 | 5.32 | 7.04 | 0.00163418 | 0.02 |
| DDIT4:ENSG00000168209_chr10:72273924-72276036_protein_coding | 1.03 | 1.43 | 7.33 | 5.81 | 0.0046363 | 0.04 |
| STMN1:ENSG00000117632_chr1:25884181-25906991_protein_coding | 0.88 | 1.43 | 5.98 | 9.58 | 0.00021026 | 0.00 |
| AL365181.2:ENSG00000272068_chr1:156637783-156641004_lncRNA | 1.06 | 1.43 | 4.32 | 4.19 | 0.01918936 | 0.10 |
| EHD3:ENSG00000013016_chr2:31234152-31269451_protein_coding | 0.18 | 1.43 | 5.04 | 4.68 | 0.01232725 | 0.07 |
| DARS2:ENSG00000117593_chr1:173824653-173858808_protein_coding | 0.97 | 1.42 | 5.22 | 29.51 | 5.09E-10 | 0.00 |
| KCTD3:ENSG00000136636_chr1:215567304-215621807_protein_coding | 1.02 | 1.42 | 5.76 | 35.88 | 1.89E-11 | 0.00 |
| PAPSS2:ENSG00000198682_chr10:87659613-87747705_protein_coding | -0.22 | 1.42 | 4.62 | 9.25 | 0.00027325 | 0.00 |
| FAM102A:ENSG00000167106_chr9:127940582-127980989_protein_coding | -0.40 | 1.41 | 6.49 | 14.07 | 7.29E-06 | 0.00 |
| ATP2B4:ENSG00000058668_chr1:203626561-203744081_protein_coding | 0.85 | 1.41 | 6.39 | 9.14 | 0.00029685 | 0.01 |
| ARHGEF2:ENSG00000116584_chr1:155946851-156007070_protein_coding | 0.80 | 1.40 | 8.08 | 25.95 | 3.72E-09 | 0.00 |
| MRPL24:ENSG00000143314_chr1:156737303-156741590_protein_coding | 0.86 | 1.40 | 6.24 | 48.63 | 5.78E-14 | 0.00 |
| CDC42SE1:ENSG00000197622_chr1:151050971-151070325_protein_coding | 0.60 | 1.40 | 7.70 | 43.88 | 4.46E-13 | 0.00 |
| ZBED6:ENSG00000257315_chr1:203795714-203854999_protein_coding | 0.60 | 1.39 | 5.53 | 19.08 | 2.43E-07 | 0.00 |
| UHMK1:ENSG00000152332_chr1:162497251-162529631_protein_coding | 0.67 | 1.38 | 7.66 | 48.53 | 6.03E-14 | 0.00 |
| MSTO1:ENSG00000125459_chr1:155610205-155614951_protein_coding | 0.82 | 1.38 | 6.72 | 30.90 | 2.41E-10 | 0.00 |
| RO60:ENSG00000116747_chr1:193059422-193091777_protein_coding | 0.57 | 1.37 | 6.10 | 49.15 | 4.65E-14 | 0.00 |
| SNRPE:ENSG00000182004_chr1:203861599-203871152_protein_coding | 0.65 | 1.36 | 6.27 | 40.83 | 1.77E-12 | 0.00 |
| ZBTB7B:ENSG00000160685_chr1:155002630-155018522_protein_coding | 0.77 | 1.36 | 6.44 | 44.64 | 3.20E-13 | 0.00 |
| GPRC5D:ENSG00000111291_chr12:12940775-12952147_protein_coding | 0.75 | 1.36 | 7.46 | 5.14 | 0.00826989 | 0.06 |
| RABIF:ENSG00000183155_chr1:202878282-202889149_protein_coding | 0.76 | 1.36 | 4.27 | 41.19 | 1.50E-12 | 0.00 |
| PLEC:ENSG00000178209_chr8:143915147-143976734_protein_coding | -0.06 | 1.36 | 8.45 | 17.37 | 7.48E-07 | 0.00 |
| CCDC86:ENSG00000110104_chr11:60842113-60851081_protein_coding | 0.54 | 1.36 | 5.03 | 12.04 | 3.20E-05 | 0.00 |
| C1orf35:ENSG00000143793_chr1:228100726-228105411_protein_coding | 0.39 | 1.35 | 4.05 | 21.60 | 4.96E-08 | 0.00 |
| TMEM9:ENSG00000116857_chr1:201134772-201171574_protein_coding | 0.94 | 1.35 | 5.80 | 22.32 | 3.19E-08 | 0.00 |
| NUSAP1:ENSG00000137804_chr15:41320794-41381050_protein_coding | 0.99 | 1.35 | 4.57 | 9.06 | 0.00031651 | 0.01 |
| MCM2:ENSG00000073111_chr3:127598410-127622436_protein_coding | 1.10 | 1.35 | 5.09 | 7.95 | 0.00077439 | 0.01 |
| SPAG5:ENSG00000076382_chr17:28577565-28599025_protein_coding | 0.86 | 1.35 | 4.49 | 16.58 | 1.28E-06 | 0.00 |
| PEA15:ENSG00000162734_chr1:160205380-160215376_protein_coding | -0.16 | 1.34 | 6.77 | 14.28 | 6.30E-06 | 0.00 |
| FLNA:ENSG00000196924_chrX:154348524-154374638_protein_coding | 0.24 | 1.34 | 7.92 | 6.23 | 0.00323803 | 0.03 |
| CACYBP:ENSG00000116161_chr1:174999163-175012027_protein_coding | 0.62 | 1.33 | 6.39 | 56.29 | 2.69E-15 | 0.00 |
| TSEN15:ENSG00000198860_chr1:184051651-184123978_protein_coding | 0.99 | 1.33 | 6.22 | 30.29 | 3.34E-10 | 0.00 |
| RAB36:ENSG00000100228_chr22:23145326-23164350_protein_coding | 0.71 | 1.32 | 5.37 | 5.93 | 0.00418622 | 0.03 |
| GPATCH4:ENSG00000160818_chr1:156594487-156601496_protein_coding | 0.89 | 1.31 | 5.72 | 36.52 | 1.38E-11 | 0.00 |
| SMYD3:ENSG00000185420_chr1:245749342-246507312_protein_coding | 0.18 | 1.31 | 4.11 | 12.13 | 3.01E-05 | 0.00 |
| AL160408.2:ENSG00000228830_chr1:234607008-234609483_lncRNA | 0.31 | 1.31 | 5.87 | 11.86 | 3.67E-05 | 0.00 |
| TIMM17A:ENSG00000134375_chr1:201955503-201970664_protein_coding | 0.87 | 1.31 | 6.53 | 49.01 | 4.93E-14 | 0.00 |
| SLC19A2:ENSG00000117479_chr1:169463909-169485944_protein_coding | 1.16 | 1.30 | 4.01 | 20.35 | 1.08E-07 | 0.00 |
| URB2:ENSG00000135763_chr1:229626247-229660200_protein_coding | 1.02 | 1.30 | 4.84 | 60.06 | 6.53E-16 | 0.00 |
| ANXA11:ENSG00000122359_chr10:80150889-80205572_protein_coding | 0.17 | 1.29 | 6.97 | 11.03 | 6.88E-05 | 0.00 |
| WHRN:ENSG00000095397_chr9:114402080-114505473_protein_coding | -0.28 | 1.29 | 6.73 | 5.56 | 0.00573888 | 0.04 |
| SNAPIN:ENSG00000143553_chr1:153658703-153661852_protein_coding | 0.66 | 1.29 | 4.61 | 41.96 | 1.06E-12 | 0.00 |
| PER1:ENSG00000179094_chr17:8140472-8156506_protein_coding | 0.13 | 1.29 | 7.49 | 4.23 | 0.01844947 | 0.10 |
| H1FX-AS1:ENSG00000206417_chr3:129315392-129326225_lncRNA | 0.78 | 1.29 | 4.00 | 7.94 | 0.00077778 | 0.01 |
| GABPB2:ENSG00000143458_chr1:151070578-151125542_protein_coding | 0.70 | 1.29 | 5.33 | 22.93 | 2.19E-08 | 0.00 |
| MRPL9:ENSG00000143436_chr1:151759647-151763496_protein_coding | 0.52 | 1.29 | 5.87 | 81.10 | 5.99E-19 | 0.00 |
| RECQL4:ENSG00000160957_chr8:144511288-144517845_protein_coding | 0.85 | 1.29 | 4.05 | 11.76 | 3.95E-05 | 0.00 |
| STING1:ENSG00000184584_chr5:139475533-139482935_protein_coding | 0.66 | 1.28 | 6.25 | 4.21 | 0.018757 | 0.10 |
| MTX1:ENSG00000173171_chr1:155208699-155213824_protein_coding | 0.60 | 1.28 | 5.52 | 52.10 | 1.39E-14 | 0.00 |
| ETV3:ENSG00000117036_chr1:157121191-157138474_protein_coding | 0.53 | 1.28 | 5.31 | 19.10 | 2.40E-07 | 0.00 |
| FBN1:ENSG00000166147_chr15:48408313-48645721_protein_coding | -1.58 | 1.28 | 4.84 | 8.77 | 0.00039987 | 0.01 |
| STX2:ENSG00000111450_chr12:130789600-130839266_protein_coding | 0.56 | 1.28 | 4.55 | 20.77 | 8.32E-08 | 0.00 |
| SYNE3:ENSG00000176438_chr14:95407266-95475836_protein_coding | 0.28 | 1.27 | 4.98 | 7.75 | 0.00091366 | 0.01 |
| MIR4435-2HG:ENSG00000172965_chr2:111006015-111523376_lncRNA | 0.51 | 1.27 | 4.46 | 5.91 | 0.00424822 | 0.03 |
| MID1IP1:ENSG00000165175_chrX:38801432-38806537_protein_coding | 1.46 | 1.27 | 4.39 | 4.18 | 0.0193284 | 0.10 |
| RALGPS2:ENSG00000116191_chr1:178725147-178921841_protein_coding | 0.62 | 1.27 | 8.39 | 15.60 | 2.50E-06 | 0.00 |
| ARID5A:ENSG00000196843_chr2:96536743-96552634_protein_coding | -0.38 | 1.27 | 7.18 | 8.22 | 0.00062092 | 0.01 |
| FH:ENSG00000091483_chr1:241497603-241519755_protein_coding | 0.73 | 1.27 | 6.30 | 55.82 | 3.22E-15 | 0.00 |
| DPM3:ENSG00000179085_chr1:155139891-155140595_protein_coding | 0.74 | 1.26 | 5.01 | 14.82 | 4.30E-06 | 0.00 |
| MCM4:ENSG00000104738_chr8:47960185-47978160_protein_coding | 0.81 | 1.26 | 5.14 | 12.12 | 3.02E-05 | 0.00 |
| TFB2M:ENSG00000162851_chr1:246540561-246566261_protein_coding | 0.79 | 1.26 | 5.09 | 54.70 | 4.99E-15 | 0.00 |
| CREB3L4:ENSG00000143578_chr1:153967534-153974361_protein_coding | 0.91 | 1.26 | 4.10 | 19.68 | 1.66E-07 | 0.00 |
| COTL1:ENSG00000103187_chr16:84565596-84618078_protein_coding | -1.05 | 1.26 | 4.10 | 5.53 | 0.00588485 | 0.04 |
| GBA:ENSG00000177628_chr1:155234452-155244699_protein_coding | 0.76 | 1.25 | 6.74 | 26.73 | 2.38E-09 | 0.00 |
| ATP8B2:ENSG00000143515_chr1:154325553-154351304_protein_coding | 0.78 | 1.25 | 9.17 | 36.36 | 1.49E-11 | 0.00 |
| PHF13:ENSG00000116273_chr1:6613731-6624030_protein_coding | 0.16 | 1.25 | 4.12 | 10.95 | 7.31E-05 | 0.00 |
| SCNM1:ENSG00000163156_chr1:151156664-151170296_protein_coding | 0.64 | 1.25 | 4.67 | 63.99 | 1.59E-16 | 0.00 |
| UBAP2L:ENSG00000143569_chr1:154220179-154271510_protein_coding | 0.60 | 1.25 | 7.43 | 74.59 | 4.50E-18 | 0.00 |
| DESI2:ENSG00000121644_chr1:244653103-244709033_protein_coding | 0.77 | 1.24 | 6.43 | 54.30 | 5.82E-15 | 0.00 |
| CORO7:ENSG00000262246_chr16:4354542-4425705_protein_coding | -0.27 | 1.24 | 6.53 | 9.71 | 0.00018937 | 0.00 |
| FAM20B:ENSG00000116199_chr1:179025804-179076567_protein_coding | 0.88 | 1.24 | 5.78 | 27.14 | 1.88E-09 | 0.00 |
| DOCK11:ENSG00000147251_chrX:118495898-118686163_protein_coding | 0.31 | 1.23 | 5.37 | 5.75 | 0.00487213 | 0.04 |
| ZBTB43:ENSG00000169155_chr9:126805006-126838210_protein_coding | -0.23 | 1.23 | 5.76 | 13.44 | 1.15E-05 | 0.00 |
| HEATR1:ENSG00000119285_chr1:236549005-236604516_protein_coding | 0.82 | 1.23 | 6.60 | 34.86 | 3.13E-11 | 0.00 |
| ADAM8:ENSG00000151651_chr10:133262420-133276868_protein_coding | -0.48 | 1.23 | 4.60 | 7.39 | 0.00123003 | 0.01 |
| NTPCR:ENSG00000135778_chr1:232950605-232983882_protein_coding | 0.71 | 1.23 | 4.44 | 31.38 | 1.87E-10 | 0.00 |
| PDCL3:ENSG00000115539_chr2:100562993-100576739_protein_coding | 0.53 | 1.23 | 4.15 | 20.35 | 1.08E-07 | 0.00 |
| SIAE:ENSG00000110013_chr11:124633113-124695707_protein_coding | 0.91 | 1.23 | 5.82 | 7.51 | 0.00110813 | 0.01 |
| TARS2:ENSG00000143374_chr1:150487364-150507609_protein_coding | 0.72 | 1.22 | 5.37 | 59.56 | 7.86E-16 | 0.00 |
| C4A:ENSG00000244731_chr6:31982057-32002681_protein_coding | 2.52 | 1.22 | 10.70 | 6.11 | 0.00359145 | 0.03 |
| FLAD1:ENSG00000160688_chr1:154983338-154993111_protein_coding | 0.77 | 1.22 | 5.86 | 54.74 | 4.91E-15 | 0.00 |
| RRP15:ENSG00000067533_chr1:218285293-218337983_protein_coding | 0.81 | 1.22 | 4.98 | 42.23 | 9.39E-13 | 0.00 |
| CREG1:ENSG00000143162_chr1:167529117-167553805_protein_coding | -0.07 | 1.22 | 7.36 | 12.96 | 1.63E-05 | 0.00 |
| DDR2:ENSG00000162733_chr1:162631373-162787405_protein_coding | 0.56 | 1.22 | 5.02 | 10.67 | 9.05E-05 | 0.00 |
| SF3B4:ENSG00000143368_chr1:149923317-149927803_protein_coding | 0.56 | 1.21 | 6.21 | 41.25 | 1.46E-12 | 0.00 |
| RCSD1:ENSG00000198771_chr1:167630093-167708696_protein_coding | 0.40 | 1.21 | 7.23 | 15.35 | 2.96E-06 | 0.00 |
| THAP2:ENSG00000173451_chr12:71664301-71680644_protein_coding | 0.51 | 1.21 | 4.28 | 9.04 | 0.00032222 | 0.01 |
| LBR:ENSG00000143815_chr1:225401502-225428925_protein_coding | 0.61 | 1.21 | 6.21 | 26.59 | 2.58E-09 | 0.00 |
| TIPRL:ENSG00000143155_chr1:168178962-168202109_protein_coding | 0.60 | 1.21 | 5.26 | 55.55 | 3.57E-15 | 0.00 |
| RAB3GAP2:ENSG00000118873_chr1:220148293-220272453_protein_coding | 0.52 | 1.21 | 6.36 | 35.06 | 2.84E-11 | 0.00 |
| DGKE:ENSG00000153933_chr17:56834107-56869567_protein_coding | 0.18 | 1.21 | 5.86 | 14.18 | 6.75E-06 | 0.00 |
| AC116366.2:ENSG00000234290_chr5:132468890-132473043_lncRNA | -0.25 | 1.20 | 5.22 | 5.95 | 0.00412418 | 0.03 |
| NTAN1:ENSG00000157045_chr16:15037854-15056079_protein_coding | 0.19 | 1.20 | 5.20 | 16.13 | 1.74E-06 | 0.00 |
| PRCC:ENSG00000143294_chr1:156750610-156800815_protein_coding | 0.86 | 1.20 | 6.44 | 39.90 | 2.74E-12 | 0.00 |
| PNP:ENSG00000198805_chr14:20468954-20477089_protein_coding | 1.17 | 1.19 | 7.04 | 8.51 | 0.00049105 | 0.01 |
| ENSA:ENSG00000143420_chr1:150600851-150629612_protein_coding | 0.39 | 1.19 | 7.91 | 55.48 | 3.68E-15 | 0.00 |
| LDHA:ENSG00000134333_chr11:18394560-18408425_protein_coding | 0.31 | 1.18 | 8.69 | 14.24 | 6.50E-06 | 0.00 |
| BOLA1:ENSG00000178096_chr1:149887890-149900798_protein_coding | 0.67 | 1.18 | 4.05 | 15.35 | 2.97E-06 | 0.00 |
| BUB1:ENSG00000169679_chr2:110637528-110678063_protein_coding | 0.92 | 1.18 | 4.61 | 8.02 | 0.00073049 | 0.01 |
| UBQLN4:ENSG00000160803_chr1:156035299-156053798_protein_coding | 0.66 | 1.18 | 6.75 | 58.02 | 1.40E-15 | 0.00 |
| SDHC:ENSG00000143252_chr1:161314381-161371964_protein_coding | 0.64 | 1.17 | 6.42 | 52.26 | 1.31E-14 | 0.00 |
| GADD45B:ENSG00000099860_chr19:2476122-2478259_protein_coding | -0.22 | 1.17 | 7.34 | 6.60 | 0.00237594 | 0.02 |
| CERS2:ENSG00000143418_chr1:150960583-150975004_protein_coding | 0.64 | 1.17 | 8.00 | 74.82 | 4.18E-18 | 0.00 |
| TTC13:ENSG00000143643_chr1:230906243-230978875_protein_coding | 0.70 | 1.17 | 5.64 | 33.73 | 5.53E-11 | 0.00 |
| CNDP2:ENSG00000133313_chr18:74495816-74523454_protein_coding | 0.88 | 1.17 | 7.73 | 14.69 | 4.71E-06 | 0.00 |
| IFI16:ENSG00000163565_chr1:158999968-159055155_protein_coding | 0.49 | 1.17 | 7.83 | 15.33 | 3.02E-06 | 0.00 |
| MRPL55:ENSG00000162910_chr1:228106679-228109312_protein_coding | 0.54 | 1.16 | 5.74 | 36.55 | 1.36E-11 | 0.00 |
| SH3BP1:ENSG00000100092_chr22:37634654-37656119_protein_coding | 0.01 | 1.16 | 4.50 | 13.51 | 1.10E-05 | 0.00 |
| PRKAB2:ENSG00000131791_chr1:147155106-147172550_protein_coding | 0.79 | 1.16 | 4.98 | 29.62 | 4.80E-10 | 0.00 |
| SNHG3:ENSG00000242125_chr1:28505980-28510892_lncRNA | 1.25 | 1.16 | 6.02 | 11.01 | 6.99E-05 | 0.00 |
| C4B:ENSG00000224389_chr6:32014795-32035418_protein_coding | 2.62 | 1.16 | 11.05 | 7.33 | 0.0012918 | 0.01 |
| FDPS:ENSG00000160752_chr1:155308748-155320666_protein_coding | 0.69 | 1.15 | 7.09 | 75.52 | 3.36E-18 | 0.00 |
| AL390728.4:ENSG00000227671_chr1:247189851-247210856_transcribed_unprocessed_pseudogene | 0.47 | 1.15 | 6.89 | 13.52 | 1.08E-05 | 0.00 |
| TSPO:ENSG00000100300_chr22:43151547-43163242_protein_coding | -0.28 | 1.15 | 5.08 | 7.66 | 0.00098454 | 0.01 |
| PSMD4:ENSG00000159352_chr1:151254709-151267479_protein_coding | 0.62 | 1.15 | 7.12 | 57.20 | 1.90E-15 | 0.00 |
| IRF2BP2:ENSG00000168264_chr1:234604269-234609525_protein_coding | 0.32 | 1.15 | 8.05 | 10.62 | 9.36E-05 | 0.00 |
| GPR89B:ENSG00000188092_chr1:147928393-147993592_protein_coding | 0.61 | 1.15 | 5.23 | 38.79 | 4.61E-12 | 0.00 |
| HSPB1:ENSG00000106211_chr7:76302673-76304295_protein_coding | 0.01 | 1.15 | 7.35 | 8.47 | 0.00050733 | 0.01 |
| CRTC2:ENSG00000160741_chr1:153947669-153958615_protein_coding | 0.50 | 1.15 | 6.46 | 32.67 | 9.54E-11 | 0.00 |
| ANP32E:ENSG00000143401_chr1:150218417-150236156_protein_coding | 0.47 | 1.15 | 6.84 | 42.99 | 6.65E-13 | 0.00 |
| ARL8A:ENSG00000143862_chr1:202133404-202144743_protein_coding | 0.43 | 1.15 | 5.44 | 20.67 | 8.86E-08 | 0.00 |
| POGK:ENSG00000143157_chr1:166839447-166856344_protein_coding | 0.57 | 1.14 | 6.46 | 49.90 | 3.41E-14 | 0.00 |
| ZNF687:ENSG00000143373_chr1:151281618-151292176_protein_coding | 0.39 | 1.14 | 6.25 | 28.74 | 7.73E-10 | 0.00 |
| NCAPG2:ENSG00000146918_chr7:158631169-158704804_protein_coding | 0.88 | 1.14 | 4.37 | 10.34 | 0.00011591 | 0.00 |
| KRTCAP2:ENSG00000163463_chr1:155169408-155173475_protein_coding | 0.53 | 1.13 | 8.24 | 16.47 | 1.37E-06 | 0.00 |
| COX20:ENSG00000203667_chr1:244835616-244845057_protein_coding | 0.58 | 1.13 | 6.50 | 35.75 | 2.01E-11 | 0.00 |
| CTPS1:ENSG00000171793_chr1:40979688-41012565_protein_coding | 0.88 | 1.12 | 4.93 | 17.18 | 8.50E-07 | 0.00 |
| IARS2:ENSG00000067704_chr1:220094132-220148041_protein_coding | 0.62 | 1.11 | 7.02 | 33.49 | 6.27E-11 | 0.00 |
| CCDC6:ENSG00000108091_chr10:59788747-59906556_protein_coding | 0.47 | 1.11 | 6.94 | 5.02 | 0.00914417 | 0.06 |
| LRRFIP1:ENSG00000124831_chr2:237627587-237813682_protein_coding | -0.29 | 1.11 | 6.65 | 10.75 | 8.49E-05 | 0.00 |
| STX6:ENSG00000135823_chr1:180972712-181023121_protein_coding | 0.52 | 1.11 | 4.63 | 27.30 | 1.72E-09 | 0.00 |
| PHF19:ENSG00000119403_chr9:120855651-120894896_protein_coding | 0.63 | 1.11 | 4.17 | 5.99 | 0.0039636 | 0.03 |
| ARF1:ENSG00000143761_chr1:228082660-228099212_protein_coding | 0.50 | 1.10 | 9.40 | 35.58 | 2.19E-11 | 0.00 |
| SCAMP3:ENSG00000116521_chr1:155255979-155262430_protein_coding | 0.60 | 1.10 | 6.84 | 49.35 | 4.28E-14 | 0.00 |
| NENF:ENSG00000117691_chr1:212432920-212446379_protein_coding | 0.51 | 1.10 | 5.86 | 17.30 | 7.86E-07 | 0.00 |
| YWHAH:ENSG00000128245_chr22:31944522-31957603_protein_coding | 0.03 | 1.10 | 5.78 | 17.60 | 6.44E-07 | 0.00 |
| NDUFS2:ENSG00000158864_chr1:161197104-161214395_protein_coding | 0.62 | 1.10 | 6.58 | 38.96 | 4.27E-12 | 0.00 |
| PSMB4:ENSG00000159377_chr1:151399560-151401937_protein_coding | 0.47 | 1.10 | 8.34 | 45.83 | 1.90E-13 | 0.00 |
| CORO1A:ENSG00000102879_chr16:30182827-30189076_protein_coding | 0.20 | 1.09 | 7.90 | 8.32 | 0.00057127 | 0.01 |
| TMEM183A:ENSG00000163444_chr1:203007374-203024848_protein_coding | 0.50 | 1.09 | 6.39 | 67.86 | 4.14E-17 | 0.00 |
| DEDD:ENSG00000158796_chr1:161120974-161132688_protein_coding | 0.55 | 1.09 | 5.34 | 42.84 | 7.12E-13 | 0.00 |
| PLSCR3:ENSG00000187838_chr17:7389727-7394842_protein_coding | 0.27 | 1.09 | 4.61 | 11.45 | 4.99E-05 | 0.00 |
| DUSP12:ENSG00000081721_chr1:161749758-161757238_protein_coding | 0.49 | 1.09 | 5.18 | 31.90 | 1.42E-10 | 0.00 |
| NUP133:ENSG00000069248_chr1:229440259-229508341_protein_coding | 0.73 | 1.09 | 5.93 | 37.17 | 1.00E-11 | 0.00 |
| ARNT:ENSG00000143437_chr1:150809713-150876708_protein_coding | 0.50 | 1.09 | 5.66 | 41.28 | 1.44E-12 | 0.00 |
| ZNF692:ENSG00000171163_chr1:248850006-248859144_protein_coding | 0.86 | 1.08 | 6.13 | 20.34 | 1.09E-07 | 0.00 |
| NSDHL:ENSG00000147383_chrX:152830967-152869729_protein_coding | 0.66 | 1.08 | 4.03 | 13.58 | 1.04E-05 | 0.00 |
| UTP25:ENSG00000117597_chr1:209827972-209857565_protein_coding | 0.77 | 1.08 | 5.11 | 19.04 | 2.50E-07 | 0.00 |
| DAP3:ENSG00000132676_chr1:155687960-155739010_protein_coding | 0.52 | 1.08 | 7.45 | 57.12 | 1.96E-15 | 0.00 |
| ACP6:ENSG00000162836_chr1:147629652-147670524_protein_coding | 0.60 | 1.08 | 4.67 | 10.63 | 9.33E-05 | 0.00 |
| ABHD12:ENSG00000100997_chr20:25294742-25390835_protein_coding | 0.19 | 1.08 | 6.20 | 6.61 | 0.00234386 | 0.02 |
| DENND1B:ENSG00000213047_chr1:197504748-197775696_protein_coding | 0.00 | 1.08 | 6.92 | 15.20 | 3.29E-06 | 0.00 |
| CAMSAP2:ENSG00000118200_chr1:200739558-200860704_protein_coding | 0.52 | 1.08 | 5.75 | 16.47 | 1.37E-06 | 0.00 |
| TMCO1:ENSG00000143183_chr1:165724293-165827755_protein_coding | 0.49 | 1.07 | 7.90 | 25.43 | 5.01E-09 | 0.00 |
| ILF2:ENSG00000143621_chr1:153661788-153671028_protein_coding | 0.60 | 1.07 | 7.48 | 37.62 | 8.06E-12 | 0.00 |
| SYNGAP1:ENSG00000197283_chr6:33419661-33453689_protein_coding | 0.44 | 1.07 | 4.84 | 18.48 | 3.60E-07 | 0.00 |
| APH1A:ENSG00000117362_chr1:150265399-150269580_protein_coding | 0.37 | 1.07 | 7.56 | 34.96 | 2.98E-11 | 0.00 |
| RRAGC:ENSG00000116954_chr1:38838198-38859772_protein_coding | -0.08 | 1.07 | 5.29 | 9.46 | 0.00023088 | 0.00 |
| ENO3:ENSG00000108515_chr17:4948092-4957131_protein_coding | 0.49 | 1.07 | 4.31 | 7.98 | 0.00075583 | 0.01 |
| RBM34:ENSG00000188739_chr1:235131183-235161283_protein_coding | 0.50 | 1.07 | 5.41 | 41.08 | 1.59E-12 | 0.00 |
| ACBD3:ENSG00000182827_chr1:226144679-226186741_protein_coding | 0.56 | 1.06 | 7.22 | 30.30 | 3.32E-10 | 0.00 |
| MPC2:ENSG00000143158_chr1:167916675-167937072_protein_coding | 0.32 | 1.06 | 5.81 | 43.26 | 5.90E-13 | 0.00 |
| TRIM11:ENSG00000154370_chr1:228393673-228406835_protein_coding | 0.67 | 1.06 | 4.93 | 22.15 | 3.54E-08 | 0.00 |
| SREBF1:ENSG00000072310_chr17:17810399-17837011_protein_coding | 0.15 | 1.06 | 5.15 | 6.51 | 0.00255211 | 0.02 |
| SLC25A44:ENSG00000160785_chr1:156193932-156212796_protein_coding | 0.57 | 1.06 | 5.69 | 24.43 | 8.95E-09 | 0.00 |
| MYBL2:ENSG00000101057_chr20:43667019-43716495_protein_coding | 0.70 | 1.05 | 4.74 | 7.35 | 0.00126651 | 0.01 |
| EZH2:ENSG00000106462_chr7:148807383-148884321_protein_coding | 0.90 | 1.05 | 4.43 | 11.80 | 3.83E-05 | 0.00 |
| TNIP2:ENSG00000168884_chr4:2741648-2756342_protein_coding | -0.09 | 1.05 | 4.67 | 10.10 | 0.00013991 | 0.00 |
| BAK1:ENSG00000030110_chr6:33572547-33580293_protein_coding | 0.48 | 1.05 | 5.65 | 16.36 | 1.48E-06 | 0.00 |
| LMNB1:ENSG00000113368_chr5:126776623-126837020_protein_coding | 0.40 | 1.05 | 4.59 | 11.03 | 6.85E-05 | 0.00 |
| PPOX:ENSG00000143224_chr1:161166056-161178013_protein_coding | 0.74 | 1.05 | 5.56 | 20.15 | 1.23E-07 | 0.00 |
| VPS72:ENSG00000163159_chr1:151176304-151195321_protein_coding | 0.62 | 1.05 | 5.35 | 37.22 | 9.80E-12 | 0.00 |
| ITGB1:ENSG00000150093_chr10:32900318-33005792_protein_coding | -0.17 | 1.05 | 7.48 | 5.20 | 0.00784813 | 0.05 |
| CLK2:ENSG00000176444_chr1:155262868-155278491_protein_coding | 0.64 | 1.05 | 6.62 | 34.62 | 3.54E-11 | 0.00 |
| PFDN2:ENSG00000143256_chr1:161100556-161118055_protein_coding | 0.60 | 1.05 | 4.91 | 38.45 | 5.43E-12 | 0.00 |
| MDM4:ENSG00000198625_chr1:204516379-204558120_protein_coding | 0.21 | 1.05 | 7.43 | 18.30 | 4.04E-07 | 0.00 |
| YOD1:ENSG00000180667_chr1:207043849-207052980_protein_coding | 0.23 | 1.05 | 6.04 | 8.53 | 0.00048568 | 0.01 |
| LGALS3:ENSG00000131981_chr14:55124110-55145423_protein_coding | -0.18 | 1.05 | 5.43 | 7.00 | 0.00169702 | 0.02 |
| KCNN3:ENSG00000143603_chr1:154697455-154870281_protein_coding | 0.62 | 1.04 | 7.57 | 9.01 | 0.00032868 | 0.01 |
| EIF4A3:ENSG00000141543_chr17:80134369-80147151_protein_coding | 0.41 | 1.04 | 6.00 | 10.87 | 7.72E-05 | 0.00 |
| YY1AP1:ENSG00000163374_chr1:155659443-155689000_protein_coding | 0.44 | 1.04 | 7.02 | 63.79 | 1.70E-16 | 0.00 |
| KPNA2:ENSG00000182481_chr17:68035708-68046854_protein_coding | 0.62 | 1.04 | 5.94 | 13.91 | 8.23E-06 | 0.00 |
| SSBP4:ENSG00000130511_chr19:18418864-18434562_protein_coding | 0.58 | 1.04 | 4.34 | 6.34 | 0.00294649 | 0.03 |
| BCL9:ENSG00000116128_chr1:147541501-147626216_protein_coding | 0.55 | 1.04 | 6.37 | 10.05 | 0.00014617 | 0.00 |
| MEF2D:ENSG00000116604_chr1:156463727-156500779_protein_coding | 0.65 | 1.04 | 7.91 | 22.33 | 3.17E-08 | 0.00 |
| MGAT5:ENSG00000152127_chr2:134119983-134454621_protein_coding | -0.10 | 1.03 | 5.31 | 4.39 | 0.01603467 | 0.09 |
| MIR22HG:ENSG00000186594_chr17:1711493-1717174_lncRNA | 0.27 | 1.03 | 5.42 | 4.28 | 0.01757921 | 0.10 |
| PYGO2:ENSG00000163348_chr1:154957026-154963853_protein_coding | 0.49 | 1.03 | 6.65 | 40.77 | 1.83E-12 | 0.00 |
| ADAM9:ENSG00000168615_chr8:38996869-39105261_protein_coding | 0.55 | 1.03 | 5.26 | 4.25 | 0.01803832 | 0.10 |
| ITPK1:ENSG00000100605_chr14:92936914-93116320_protein_coding | 0.72 | 1.03 | 5.18 | 10.51 | 0.00010179 | 0.00 |
| RBFA:ENSG00000101546_chr18:80034389-80050651_protein_coding | 0.73 | 1.03 | 4.50 | 15.16 | 3.40E-06 | 0.00 |
| RRP12:ENSG00000052749_chr10:97356358-97426076_protein_coding | 0.53 | 1.02 | 6.63 | 7.28 | 0.00134688 | 0.01 |
| ADAR:ENSG00000160710_chr1:154582057-154628013_protein_coding | 0.49 | 1.02 | 9.74 | 26.81 | 2.28E-09 | 0.00 |
| EDEM3:ENSG00000116406_chr1:184690237-184754907_protein_coding | 0.87 | 1.02 | 7.80 | 9.73 | 0.00018632 | 0.00 |
| TSTD1:ENSG00000215845_chr1:161037631-161038977_protein_coding | 0.52 | 1.02 | 5.70 | 14.63 | 4.91E-06 | 0.00 |
| EPHX1:ENSG00000143819_chr1:225810092-225845563_protein_coding | 0.09 | 1.02 | 5.62 | 12.75 | 1.90E-05 | 0.00 |
| UFC1:ENSG00000143222_chr1:161152776-161158856_protein_coding | 0.45 | 1.02 | 7.50 | 30.75 | 2.62E-10 | 0.00 |
| SH3BP5L:ENSG00000175137_chr1:248810446-248825915_protein_coding | 0.60 | 1.02 | 5.33 | 21.40 | 5.62E-08 | 0.00 |
| ELK4:ENSG00000158711_chr1:205597556-205632011_protein_coding | 0.48 | 1.02 | 6.64 | 25.11 | 6.01E-09 | 0.00 |
| ADSS2:ENSG00000035687_chr1:244408494-244451909_protein_coding | 0.71 | 1.02 | 6.26 | 19.12 | 2.37E-07 | 0.00 |
| CDKN2A:ENSG00000147889_chr9:21967753-21995301_protein_coding | 0.32 | 1.02 | 4.23 | 8.95 | 0.00034457 | 0.01 |
| PIGC:ENSG00000135845_chr1:172370189-172444086_protein_coding | 0.60 | 1.01 | 5.25 | 34.02 | 4.78E-11 | 0.00 |
| ABCB10:ENSG00000135776_chr1:229516582-229558707_protein_coding | 0.40 | 1.01 | 5.18 | 36.50 | 1.39E-11 | 0.00 |
| GUK1:ENSG00000143774_chr1:228139962-228148984_protein_coding | 0.38 | 1.01 | 7.59 | 30.60 | 2.84E-10 | 0.00 |
| RIT1:ENSG00000143622_chr1:155897808-155911404_protein_coding | 0.17 | 1.01 | 4.92 | 19.55 | 1.81E-07 | 0.00 |
| TOMM20:ENSG00000173726_chr1:235109341-235128837_protein_coding | 0.30 | 1.01 | 8.33 | 26.81 | 2.27E-09 | 0.00 |
| DPY19L1:ENSG00000173852_chr7:34928876-35038271_protein_coding | 0.86 | 1.01 | 6.06 | 10.23 | 0.00012675 | 0.00 |
| IZUMO4:ENSG00000099840_chr19:2096429-2099593_protein_coding | 0.06 | 1.01 | 4.35 | 6.07 | 0.00370877 | 0.03 |
| PIP5K1A:ENSG00000143398_chr1:151197949-151249536_protein_coding | 0.57 | 1.01 | 6.37 | 35.54 | 2.23E-11 | 0.00 |
| KIAA0895L:ENSG00000196123_chr16:67175599-67184040_protein_coding | 0.55 | 1.00 | 4.57 | 6.77 | 0.00205952 | 0.02 |
| AKAP1:ENSG00000121057_chr17:57085092-57121346_protein_coding | 0.69 | 1.00 | 6.14 | 13.32 | 1.26E-05 | 0.00 |
| UBE2Q1:ENSG00000160714_chr1:154548577-154559028_protein_coding | 0.43 | 1.00 | 7.17 | 49.71 | 3.70E-14 | 0.00 |
| BCL3:ENSG00000069399_chr19:44747705-44760044_protein_coding | 0.24 | 1.00 | 4.90 | 5.31 | 0.00712905 | 0.05 |
| BIK:ENSG00000100290_chr22:43110750-43129712_protein_coding | -0.25 | 1.00 | 4.18 | 5.20 | 0.0078309 | 0.05 |
| STS:ENSG00000101846_chrX:7147237-7804358_protein_coding | 1.23 | 1.00 | 5.69 | 4.35 | 0.01652956 | 0.09 |
| TADA1:ENSG00000152382_chr1:166856510-166876264_protein_coding | 0.48 | 1.00 | 4.38 | 30.61 | 2.81E-10 | 0.00 |
| KIF22:ENSG00000079616_chr16:29790719-29805384_protein_coding | 0.41 | 1.00 | 5.36 | 14.60 | 5.03E-06 | 0.00 |
| DCAF8:ENSG00000132716_chr1:160215715-160262549_protein_coding | 0.61 | 1.00 | 8.45 | 30.91 | 2.40E-10 | 0.00 |
| AL365205.1:ENSG00000124593_chr6:41780349-41790141_protein_coding | 6.37 | 0.67 | 7.06 | 44.75 | 3.05E-13 | 0.00 |
| CCND3:ENSG00000112576_chr6:41934934-42050357_protein_coding | 2.53 | 0.64 | 6.78 | 12.57 | 2.16E-05 | 0.00 |
| USP49:ENSG00000164663_chr6:41789896-41895361_protein_coding | 1.84 | 0.71 | 4.98 | 10.45 | 0.00010671 | 0.00 |
| CHAC1:ENSG00000128965_chr15:40952962-40956512_protein_coding | 1.20 | -0.12 | 5.12 | 5.60 | 0.00556117 | 0.04 |
| MYC:ENSG00000136997_chr8:127735434-127742951_protein_coding | 1.15 | 0.99 | 7.74 | 5.33 | 0.00697641 | 0.05 |
| DIPK2A:ENSG00000181744_chr3:143971823-144048719_protein_coding | 1.07 | 0.52 | 5.01 | 8.60 | 0.00045585 | 0.01 |
| WARS1:ENSG00000140105_chr14:100333790-100376805_protein_coding | 1.04 | 0.97 | 8.80 | 4.56 | 0.01381338 | 0.08 |
| LRP8:ENSG00000157193_chr1:53242364-53328469_protein_coding | 1.02 | 0.84 | 4.19 | 7.24 | 0.00138173 | 0.02 |
| TOMM6:ENSG00000214736_chr6:41787662-41789898_protein_coding | 1.01 | 0.29 | 6.85 | 8.79 | 0.00039335 | 0.01 |
| TAP1:ENSG00000168394_chr6:32845209-32853978_protein_coding | 1.00 | 0.72 | 8.61 | 5.38 | 0.00670105 | 0.05 |
| RELB:ENSG00000104856_chr19:45001449-45038198_protein_coding | -1.00 | 0.40 | 5.38 | 7.63 | 0.0010041 | 0.01 |
| CTSB:ENSG00000164733_chr8:11842524-11869448_protein_coding | -1.03 | -0.31 | 7.55 | 5.98 | 0.00399225 | 0.03 |
| EPSTI1:ENSG00000133106_chr13:42886388-42992271_protein_coding | -1.07 | -0.88 | 4.68 | 4.42 | 0.01558575 | 0.09 |
| EPB41L2:ENSG00000079819_chr6:130839347-131063322_protein_coding | -1.15 | -0.27 | 4.80 | 5.96 | 0.00407331 | 0.03 |
| ATF3:ENSG00000162772_chr1:212565334-212620777_protein_coding | -1.35 | -0.90 | 8.03 | 5.40 | 0.00656972 | 0.05 |
| CORO1C:ENSG00000110880_chr12:108645109-108731596_protein_coding | -1.55 | -0.47 | 7.38 | 6.89 | 0.00185503 | 0.02 |
| CD5L:ENSG00000073754_chr1:157830911-157898256_protein_coding | -1.95 | -0.82 | 4.60 | 6.46 | 0.00267033 | 0.02 |
| MS4A1:ENSG00000156738_chr11:60455846-60470752_protein_coding | -3.44 | 0.82 | 8.14 | 5.62 | 0.00544066 | 0.04 |
| RN7SKP176:ENSG00000260682_chr16:81961926-81962243_misc_RNA | -0.73 | -1.00 | 5.09 | 6.55 | 0.00247739 | 0.02 |
| EHD4:ENSG00000103966_chr15:41895933-41972557_protein_coding | -0.67 | -1.00 | 5.53 | 4.80 | 0.01114226 | 0.07 |
| SELENOP:ENSG00000250722_chr5:42799880-42887392_protein_coding | -0.94 | -1.01 | 6.54 | 4.89 | 0.01025076 | 0.06 |
| FKBP14:ENSG00000106080_chr7:30010587-30026702_protein_coding | -0.37 | -1.01 | 6.07 | 10.88 | 7.68E-05 | 0.00 |
| RB1:ENSG00000139687_chr13:48303726-48599436_protein_coding | -0.71 | -1.01 | 6.38 | 5.55 | 0.00580317 | 0.04 |
| SYTL1:ENSG00000142765_chr1:27342020-27353937_protein_coding | -0.44 | -1.02 | 6.61 | 9.35 | 0.00025273 | 0.00 |
| POGLUT3:ENSG00000178202_chr11:108472112-108498384_protein_coding | -0.25 | -1.02 | 5.32 | 6.24 | 0.00320191 | 0.03 |
| APH1B:ENSG00000138613_chr15:63276018-63309126_protein_coding | -0.05 | -1.02 | 4.85 | 9.07 | 0.00031464 | 0.01 |
| PRCP:ENSG00000137509_chr11:82822936-82970584_protein_coding | -0.56 | -1.02 | 7.49 | 13.24 | 1.33E-05 | 0.00 |
| DDX58:ENSG00000107201_chr9:32455302-32526208_protein_coding | -0.40 | -1.02 | 4.99 | 6.72 | 0.00213234 | 0.02 |
| EML5:ENSG00000165521_chr14:88612431-88792752_protein_coding | -0.71 | -1.02 | 4.54 | 8.50 | 0.00049638 | 0.01 |
| PDCD4:ENSG00000150593_chr10:110871795-110900006_protein_coding | -0.96 | -1.03 | 7.56 | 10.65 | 9.19E-05 | 0.00 |
| FOXP2:ENSG00000128573_chr7:114086327-114693772_protein_coding | -0.40 | -1.03 | 4.74 | 7.84 | 0.0008443 | 0.01 |
| DUSP1:ENSG00000120129_chr5:172768096-172771195_protein_coding | -0.57 | -1.03 | 10.92 | 6.57 | 0.00243681 | 0.02 |
| PLCG2:ENSG00000197943_chr16:81739097-81962685_protein_coding | -0.57 | -1.03 | 7.74 | 13.34 | 1.23E-05 | 0.00 |
| SLC17A9:ENSG00000101194_chr20:62952707-62969585_protein_coding | -0.37 | -1.03 | 7.14 | 8.45 | 0.00051769 | 0.01 |
| RAPGEF2:ENSG00000109756_chr4:159103013-159360174_protein_coding | -0.31 | -1.04 | 8.03 | 8.44 | 0.00052129 | 0.01 |
| ATXN1:ENSG00000124788_chr6:16299112-16761491_protein_coding | -0.76 | -1.04 | 6.54 | 9.89 | 0.00016439 | 0.00 |
| LPXN:ENSG00000110031_chr11:58526871-58578220_protein_coding | -0.48 | -1.04 | 5.74 | 6.89 | 0.00185816 | 0.02 |
| SC5D:ENSG00000109929_chr11:121292681-121313410_protein_coding | -0.67 | -1.04 | 4.72 | 8.60 | 0.00045595 | 0.01 |
| CERCAM:ENSG00000167123_chr9:128411751-128437351_protein_coding | -0.09 | -1.04 | 6.78 | 5.53 | 0.00591202 | 0.04 |
| TXLNB:ENSG00000164440_chr6:139240061-139291998_protein_coding | -0.37 | -1.04 | 4.72 | 4.27 | 0.01771804 | 0.10 |
| USPL1:ENSG00000132952_chr13:30617693-30660770_protein_coding | -0.63 | -1.05 | 5.26 | 10.25 | 0.00012438 | 0.00 |
| SEL1L3:ENSG00000091490_chr4:25747433-25863760_protein_coding | -0.33 | -1.06 | 9.52 | 6.28 | 0.00310762 | 0.03 |
| KIF13B:ENSG00000197892_chr8:29067278-29263124_protein_coding | -0.71 | -1.06 | 6.34 | 17.86 | 5.43E-07 | 0.00 |
| ITSN1:ENSG00000205726_chr21:33642400-33899861_protein_coding | -0.77 | -1.06 | 5.29 | 5.97 | 0.00405327 | 0.03 |
| RAPGEF3:ENSG00000079337_chr12:47734363-47771040_protein_coding | -1.40 | -1.06 | 5.16 | 9.37 | 0.00024764 | 0.00 |
| EAF2:ENSG00000145088_chr3:121835183-121886526_protein_coding | -0.47 | -1.06 | 7.37 | 7.18 | 0.00145546 | 0.02 |
| IL16:ENSG00000172349_chr15:81159575-81314058_protein_coding | -0.43 | -1.09 | 7.59 | 9.31 | 0.00025941 | 0.00 |
| CD40:ENSG00000101017_chr20:46118278-46129863_protein_coding | -0.89 | -1.10 | 4.85 | 11.03 | 6.88E-05 | 0.00 |
| CSNK1G3:ENSG00000151292_chr5:123512099-123617045_protein_coding | -0.51 | -1.11 | 6.45 | 14.41 | 5.75E-06 | 0.00 |
| ACVR1C:ENSG00000123612_chr2:157526767-157628864_protein_coding | -0.17 | -1.11 | 4.44 | 4.63 | 0.01289237 | 0.08 |
| EPB41L4A:ENSG00000129595_chr5:112142441-112419313_protein_coding | -0.73 | -1.11 | 5.44 | 12.48 | 2.31E-05 | 0.00 |
| ARHGAP25:ENSG00000163219_chr2:68679601-68826833_protein_coding | -0.52 | -1.12 | 5.77 | 15.30 | 3.08E-06 | 0.00 |
| KLF10:ENSG00000155090_chr8:102648784-102655725_protein_coding | -0.82 | -1.13 | 7.40 | 5.45 | 0.00630533 | 0.05 |
| SLC35F2:ENSG00000110660_chr11:107790991-107928293_protein_coding | -0.57 | -1.13 | 5.78 | 14.11 | 7.11E-06 | 0.00 |
| RAB30:ENSG00000137502_chr11:82973133-83071923_protein_coding | -0.86 | -1.14 | 7.75 | 8.09 | 0.00068876 | 0.01 |
| PCED1B:ENSG00000179715_chr12:47079603-47236662_protein_coding | -0.36 | -1.14 | 4.28 | 4.24 | 0.01826404 | 0.10 |
| SP140:ENSG00000079263_chr2:230203110-230313215_protein_coding | -0.54 | -1.14 | 5.92 | 11.69 | 4.16E-05 | 0.00 |
| APOL1:ENSG00000100342_chr22:36253071-36267530_protein_coding | -0.93 | -1.16 | 6.63 | 9.33 | 0.00025653 | 0.00 |
| ZNF215:ENSG00000149054_chr11:6926404-7001004_protein_coding | -0.47 | -1.17 | 6.21 | 10.30 | 0.00012014 | 0.00 |
| ADA2:ENSG00000093072_chr22:17178790-17258235_protein_coding | -0.76 | -1.17 | 9.04 | 6.56 | 0.00245178 | 0.02 |
| SLC15A2:ENSG00000163406_chr3:121894401-121944188_protein_coding | -0.42 | -1.18 | 5.51 | 8.70 | 0.00042363 | 0.01 |
| IQCN:ENSG00000130518_chr19:18257097-18274509_protein_coding | -1.64 | -1.19 | 4.01 | 4.84 | 0.01074039 | 0.07 |
| AL162581.1:ENSG00000202198_chr6:52995620-52995950_misc_RNA | -0.65 | -1.19 | 4.13 | 4.34 | 0.01669627 | 0.09 |
| FBXW7:ENSG00000109670_chr4:152320544-152536092_protein_coding | -0.54 | -1.19 | 8.78 | 16.42 | 1.43E-06 | 0.00 |
| AC078883.1:ENSG00000225205_chr2:172480840-172556596_lncRNA | -0.49 | -1.20 | 4.77 | 12.78 | 1.85E-05 | 0.00 |
| LRP1:ENSG00000123384_chr12:57128483-57213361_protein_coding | -0.72 | -1.21 | 5.86 | 6.47 | 0.00264567 | 0.02 |
| HS2ST1:ENSG00000153936_chr1:86914635-87109982_protein_coding | -0.84 | -1.22 | 6.48 | 16.54 | 1.31E-06 | 0.00 |
| MRPS31:ENSG00000102738_chr13:40729128-40771190_protein_coding | -0.94 | -1.22 | 5.23 | 15.57 | 2.54E-06 | 0.00 |
| JUN:ENSG00000177606_chr1:58780791-58784047_protein_coding | -0.85 | -1.22 | 12.04 | 9.82 | 0.00017381 | 0.00 |
| SCAPER:ENSG00000140386_chr15:76347904-76905444_protein_coding | -0.74 | -1.22 | 5.42 | 21.92 | 4.06E-08 | 0.00 |
| FCHSD2:ENSG00000137478_chr11:72836745-73142318_protein_coding | -0.73 | -1.23 | 7.67 | 15.13 | 3.47E-06 | 0.00 |
| SASH3:ENSG00000122122_chrX:129779949-129795201_protein_coding | -0.54 | -1.24 | 5.92 | 5.30 | 0.00720451 | 0.05 |
| CASP4:ENSG00000196954_chr11:104942866-104969366_protein_coding | -0.57 | -1.25 | 6.37 | 10.81 | 8.10E-05 | 0.00 |
| BCAR3:ENSG00000137936_chr1:93561741-93847150_protein_coding | -1.25 | -1.25 | 4.72 | 9.52 | 0.00022102 | 0.00 |
| CAMK2D:ENSG00000145349_chr4:113451032-113761927_protein_coding | -0.36 | -1.26 | 5.65 | 7.82 | 0.00086299 | 0.01 |
| RCBTB2:ENSG00000136161_chr13:48488959-48533256_protein_coding | -0.50 | -1.26 | 5.61 | 5.99 | 0.00396953 | 0.03 |
| DRAM1:ENSG00000136048_chr12:101877580-102012130_protein_coding | -1.07 | -1.28 | 4.01 | 11.57 | 4.55E-05 | 0.00 |
| XRCC4:ENSG00000152422_chr5:83077498-83353787_protein_coding | -0.71 | -1.29 | 4.73 | 10.87 | 7.77E-05 | 0.00 |
| TLR6:ENSG00000174130_chr4:38823715-38856817_protein_coding | -0.73 | -1.29 | 5.29 | 13.87 | 8.45E-06 | 0.00 |
| ACACB:ENSG00000076555_chr12:109116595-109268226_protein_coding | -0.67 | -1.30 | 5.24 | 6.69 | 0.0021976 | 0.02 |
| PIK3AP1:ENSG00000155629_chr10:96593315-96720514_protein_coding | -1.48 | -1.31 | 5.50 | 10.43 | 0.00010825 | 0.00 |
| SLC40A1:ENSG00000138449_chr2:189560590-189583758_protein_coding | -1.59 | -1.32 | 5.70 | 8.55 | 0.00047621 | 0.01 |
| NCOA7:ENSG00000111912_chr6:125781161-125932034_protein_coding | -0.39 | -1.32 | 5.96 | 15.96 | 1.95E-06 | 0.00 |
| EGR1:ENSG00000120738_chr5:138465479-138469303_protein_coding | -1.01 | -1.32 | 9.63 | 4.83 | 0.01079809 | 0.07 |
| DENND6B:ENSG00000205593_chr22:50309030-50327012_protein_coding | 0.26 | -1.33 | 5.68 | 4.91 | 0.01007339 | 0.06 |
| PIF1:ENSG00000140451_chr15:64815632-64825668_protein_coding | -0.52 | -1.34 | 4.79 | 7.24 | 0.00138965 | 0.02 |
| AC007952.4:ENSG00000262202_chr17:19112000-19112636_lncRNA | -0.24 | -1.35 | 4.02 | 4.32 | 0.0170925 | 0.09 |
| TBCEL:ENSG00000154114_chr11:121024072-121090775_protein_coding | -0.99 | -1.35 | 7.28 | 8.80 | 0.00038842 | 0.01 |
| DUSP6:ENSG00000139318_chr12:89347235-89352501_protein_coding | -1.02 | -1.35 | 6.73 | 5.47 | 0.00619823 | 0.05 |
| CD68:ENSG00000129226_chr17:7579491-7582111_protein_coding | -1.87 | -1.37 | 4.95 | 5.87 | 0.00440085 | 0.04 |
| GRAMD1C:ENSG00000178075_chr3:113828182-113947174_protein_coding | -0.56 | -1.40 | 4.91 | 8.74 | 0.0004092 | 0.01 |
| AMPD1:ENSG00000116748_chr1:114673090-114695618_protein_coding | -0.39 | -1.41 | 6.86 | 5.20 | 0.00782809 | 0.05 |
| HLA-DPA1:ENSG00000231389_chr6:33064569-33080775_protein_coding | -1.89 | -1.41 | 4.25 | 6.37 | 0.00287312 | 0.03 |
| ALDH2:ENSG00000111275_chr12:111766887-111817532_protein_coding | -1.07 | -1.43 | 4.97 | 6.47 | 0.00263874 | 0.02 |
| ITGA8:ENSG00000077943_chr10:15513954-15719922_protein_coding | -0.63 | -1.44 | 8.06 | 4.24 | 0.0182205 | 0.10 |
| LILRB1:ENSG00000104972_chr19:54617158-54637528_protein_coding | -0.52 | -1.44 | 5.71 | 4.59 | 0.01333982 | 0.08 |
| ATP10D:ENSG00000145246_chr4:47485275-47593486_protein_coding | -0.45 | -1.46 | 5.78 | 16.21 | 1.64E-06 | 0.00 |
| UBA7:ENSG00000182179_chr3:49805209-49813953_protein_coding | -0.64 | -1.46 | 6.86 | 12.91 | 1.69E-05 | 0.00 |
| FAM95B1:ENSG00000223839_chr9:40321299-40329221_transcribed_unprocessed_pseudogene | -1.65 | -1.46 | 4.30 | 6.96 | 0.00175207 | 0.02 |
| ERAP2:ENSG00000164308_chr5:96875986-96919703_protein_coding | -0.80 | -1.47 | 7.74 | 7.93 | 0.00078594 | 0.01 |
| AC024940.2:ENSG00000177359_chr12:31111652-31201235_transcribed_unprocessed_pseudogene | 0.86 | -1.47 | 5.49 | 4.95 | 0.00976757 | 0.06 |
| PIP5K1B:ENSG00000107242_chr9:68705240-69009176_protein_coding | -0.89 | -1.48 | 6.53 | 12.41 | 2.44E-05 | 0.00 |
| ALOX5:ENSG00000012779_chr10:45374176-45446119_protein_coding | -0.56 | -1.50 | 6.80 | 6.56 | 0.00244293 | 0.02 |
| PRKCA:ENSG00000154229_chr17:66302613-66810743_protein_coding | -0.86 | -1.51 | 5.79 | 5.69 | 0.00513982 | 0.04 |
| TNFSF10:ENSG00000121858_chr3:172505508-172523475_protein_coding | -1.14 | -1.51 | 5.74 | 6.57 | 0.00243064 | 0.02 |
| PCED1B-AS1:ENSG00000247774_chr12:47205898-47216456_lncRNA | -0.46 | -1.54 | 4.79 | 6.65 | 0.00227064 | 0.02 |
| TMEM156:ENSG00000121895_chr4:38966744-39032922_protein_coding | -1.30 | -1.54 | 5.38 | 4.71 | 0.0120964 | 0.07 |
| SCYL2:ENSG00000136021_chr12:100267140-100341715_protein_coding | -1.19 | -1.54 | 7.46 | 11.22 | 5.92E-05 | 0.00 |
| LAMP3:ENSG00000078081_chr3:183122215-183163839_protein_coding | -1.28 | -1.55 | 6.38 | 10.70 | 8.79E-05 | 0.00 |
| GFI1:ENSG00000162676_chr1:92473043-92486925_protein_coding | -0.70 | -1.56 | 4.72 | 14.25 | 6.41E-06 | 0.00 |
| LINC02362:ENSG00000249096_chr4:184365180-184382416_lncRNA | -0.39 | -1.56 | 6.04 | 5.89 | 0.00433677 | 0.04 |
| NEDD9:ENSG00000111859_chr6:11183298-11382348_protein_coding | -1.03 | -1.56 | 6.73 | 12.56 | 2.18E-05 | 0.00 |
| LTBP1:ENSG00000049323_chr2:32946953-33399509_protein_coding | -1.16 | -1.57 | 5.60 | 10.19 | 0.00013105 | 0.00 |
| PDZRN4:ENSG00000165966_chr12:41188320-41574745_protein_coding | -0.80 | -1.62 | 4.72 | 5.00 | 0.00934176 | 0.06 |
| TLR10:ENSG00000174123_chr4:38772238-38782990_protein_coding | -0.98 | -1.65 | 5.50 | 7.52 | 0.00110434 | 0.01 |
| SIDT1:ENSG00000072858_chr3:113532296-113629578_protein_coding | -0.89 | -1.68 | 5.31 | 22.81 | 2.35E-08 | 0.00 |
| SLC46A3:ENSG00000139508_chr13:28700064-28718970_protein_coding | -1.06 | -1.69 | 4.03 | 6.51 | 0.00254925 | 0.02 |
| LGALS3BP:ENSG00000108679_chr17:78971238-78979947_protein_coding | -0.03 | -1.69 | 4.64 | 6.65 | 0.00226846 | 0.02 |
| LRRK2:ENSG00000188906_chr12:40196744-40369285_protein_coding | -1.00 | -1.72 | 5.73 | 5.68 | 0.00517123 | 0.04 |
| SLC22A17:ENSG00000092096_chr14:23346306-23352912_protein_coding | -1.87 | -1.76 | 4.74 | 4.78 | 0.01132904 | 0.07 |
| TRANK1:ENSG00000168016_chr3:36826820-36945098_protein_coding | -0.82 | -1.78 | 6.43 | 20.81 | 8.08E-08 | 0.00 |
| FAM13A:ENSG00000138640_chr4:88725955-89111398_protein_coding | -0.57 | -1.80 | 6.43 | 14.51 | 5.37E-06 | 0.00 |
| CHSY3:ENSG00000198108_chr5:129904465-130186634_protein_coding | -0.73 | -1.81 | 4.52 | 4.95 | 0.00973083 | 0.06 |
| IL5RA:ENSG00000091181_chr3:3066326-3126613_protein_coding | -1.54 | -1.82 | 5.58 | 4.96 | 0.00968559 | 0.06 |
| APOE:ENSG00000130203_chr19:44905791-44909393_protein_coding | -2.62 | -1.83 | 4.80 | 10.46 | 0.00010606 | 0.00 |
| MOXD1:ENSG00000079931_chr6:132296055-132401475_protein_coding | -0.23 | -1.83 | 6.53 | 6.69 | 0.0022016 | 0.02 |
| GPRASP1:ENSG00000198932_chrX:102651092-102659083_protein_coding | -1.71 | -1.83 | 4.42 | 6.57 | 0.00242932 | 0.02 |
| C1QB:ENSG00000173369_chr1:22652762-22661637_protein_coding | 0.74 | -1.86 | 6.14 | 4.86 | 0.01055623 | 0.07 |
| TRIM2:ENSG00000109654_chr4:153152342-153339317_protein_coding | -0.05 | -1.87 | 4.16 | 4.56 | 0.01372679 | 0.08 |
| C1QA:ENSG00000173372_chr1:22636628-22639678_protein_coding | -2.12 | -1.90 | 4.34 | 7.53 | 0.00108807 | 0.01 |
| P2RY10:ENSG00000078589_chrX:78945332-78963727_protein_coding | -1.22 | -1.91 | 4.69 | 14.67 | 4.79E-06 | 0.00 |
| C1R:ENSG00000159403_chr12:7080214-7092540_protein_coding | -1.05 | -1.93 | 5.02 | 5.85 | 0.00445548 | 0.04 |
| DAB2IP:ENSG00000136848_chr9:121567057-121785530_protein_coding | -0.61 | -1.98 | 4.31 | 6.09 | 0.00364076 | 0.03 |
| NID2:ENSG00000087303_chr14:52004803-52069228_protein_coding | 0.06 | -1.99 | 4.95 | 5.97 | 0.00405205 | 0.03 |
| ESRRG:ENSG00000196482_chr1:216503246-217137755_protein_coding | 0.13 | -2.03 | 4.16 | 9.67 | 0.00019557 | 0.00 |
| KIF19:ENSG00000196169_chr17:74326210-74355820_protein_coding | -2.07 | -2.12 | 4.57 | 9.63 | 0.00020293 | 0.00 |
| VCAM1:ENSG00000162692_chr1:100719742-100739045_protein_coding | -2.37 | -2.17 | 4.42 | 7.66 | 0.00097971 | 0.01 |
| CD163:ENSG00000177575_chr12:7470813-7503893_protein_coding | -2.43 | -2.21 | 4.64 | 8.92 | 0.00035402 | 0.01 |
| CNTN5:ENSG00000149972_chr11:99020949-100358885_protein_coding | -1.06 | -2.22 | 5.41 | 10.22 | 0.00012744 | 0.00 |
| MAP2:ENSG00000078018_chr2:209424058-209734118_protein_coding | -0.68 | -2.24 | 4.48 | 9.50 | 0.00022404 | 0.00 |
| F2R:ENSG00000181104_chr5:76716126-76735770_protein_coding | -2.43 | -2.25 | 4.80 | 5.90 | 0.00427274 | 0.03 |
| HMOX1:ENSG00000100292_chr22:35380361-35394207_protein_coding | -2.04 | -2.38 | 5.28 | 13.90 | 8.25E-06 | 0.00 |
| CD79A:ENSG00000105369_chr19:41877279-41881372_protein_coding | -0.11 | -2.41 | 7.98 | 5.36 | 0.00679762 | 0.05 |
| TNFRSF4:ENSG00000186827_chr1:1211340-1214153_protein_coding | 2.79 | -2.44 | 4.34 | 18.72 | 3.08E-07 | 0.00 |
| PAX5:ENSG00000196092_chr9:36833269-37034268_protein_coding | -0.81 | -2.50 | 4.81 | 5.75 | 0.00489409 | 0.04 |
| MYOF:ENSG00000138119_chr10:93306429-93482334_protein_coding | -1.40 | -2.65 | 4.61 | 8.56 | 0.00047447 | 0.01 |
| CTSW:ENSG00000172543_chr11:65879837-65883741_protein_coding | -1.87 | -2.68 | 4.89 | 6.67 | 0.00224122 | 0.02 |
| GRIK4:ENSG00000149403_chr11:120511746-120988906_protein_coding | -1.81 | -2.72 | 5.06 | 13.45 | 1.14E-05 | 0.00 |
| GBA3:ENSG00000249948_chr4:22692914-22819575_polymorphic_pseudogene | -2.53 | -2.82 | 5.37 | 8.57 | 0.00046861 | 0.01 |
| STAP1:ENSG00000035720_chr4:67558727-67607337_protein_coding | -1.10 | -2.84 | 5.43 | 25.39 | 5.14E-09 | 0.00 |
| RRAS2:ENSG00000133818_chr11:14277922-14364506_protein_coding | -1.37 | -2.84 | 4.34 | 6.58 | 0.00241543 | 0.02 |
| LINC01781:ENSG00000234184_chr1:80535755-80646791_lncRNA | -1.09 | -3.05 | 5.37 | 7.63 | 0.0010025 | 0.01 |
| CD69:ENSG00000110848_chr12:9752486-9760901_protein_coding | -3.13 | -3.06 | 4.41 | 8.60 | 0.00045759 | 0.01 |
| APOL3:ENSG00000128284_chr22:36140330-36166177_protein_coding | -1.96 | -3.43 | 4.63 | 20.15 | 1.23E-07 | 0.00 |
| PHLDA1:ENSG00000139289_chr12:76025447-76033932_protein_coding | -1.57 | -3.57 | 6.41 | 11.34 | 5.43E-05 | 0.00 |
| ADGRL2:ENSG00000117114_chr1:81306147-81992436_protein_coding | -0.24 | -4.35 | 4.71 | 7.75 | 0.00090955 | 0.01 |
| COL4A6:ENSG00000197565_chrX:108155607-108439497_protein_coding | -1.52 | -4.36 | 4.75 | 10.90 | 7.55E-05 | 0.00 |
| GPR15:ENSG00000154165_chr3:98531978-98534681_protein_coding | -1.96 | -8.64 | 4.44 | 14.16 | 6.87E-06 | 0.00 |

Table S5. Top deregulated pathways by ingenuity pathways analysis in Amp1q patients.


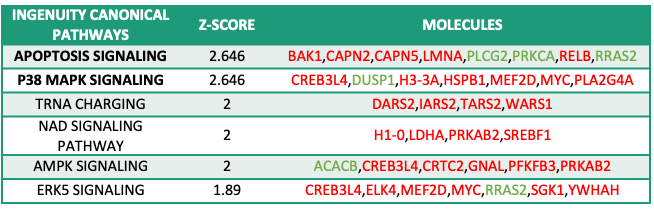


Red🡪 Up-regulated (Amp1q vs. normal 1q)

Green 🡪Down-regulated (Amp1q vs. normal 1q)

Figure S1. Amp1q clone size (% of nuclei with ≥4 copies of 1q) affects the risk of a PFS event. Epanechnikov kernel-smoothed estimated hazard rates were used to study the risk of progression or death according to the % of nuclei with ≥ 4 copies of 1q.

Figure S2. Progression-free survival from second randomization (R2) according to 1q alterations. HR values and the 95% CIs were estimated with a Cox proportional hazards model adjusted for R-ISS stage (R-ISS III vs. II vs. I vs. not available), age (≥ 60 vs <60 years) first (KRd-ASCT vs. KRd12 vs. KCd-ASCT) and second (KR vs R) randomization arm.


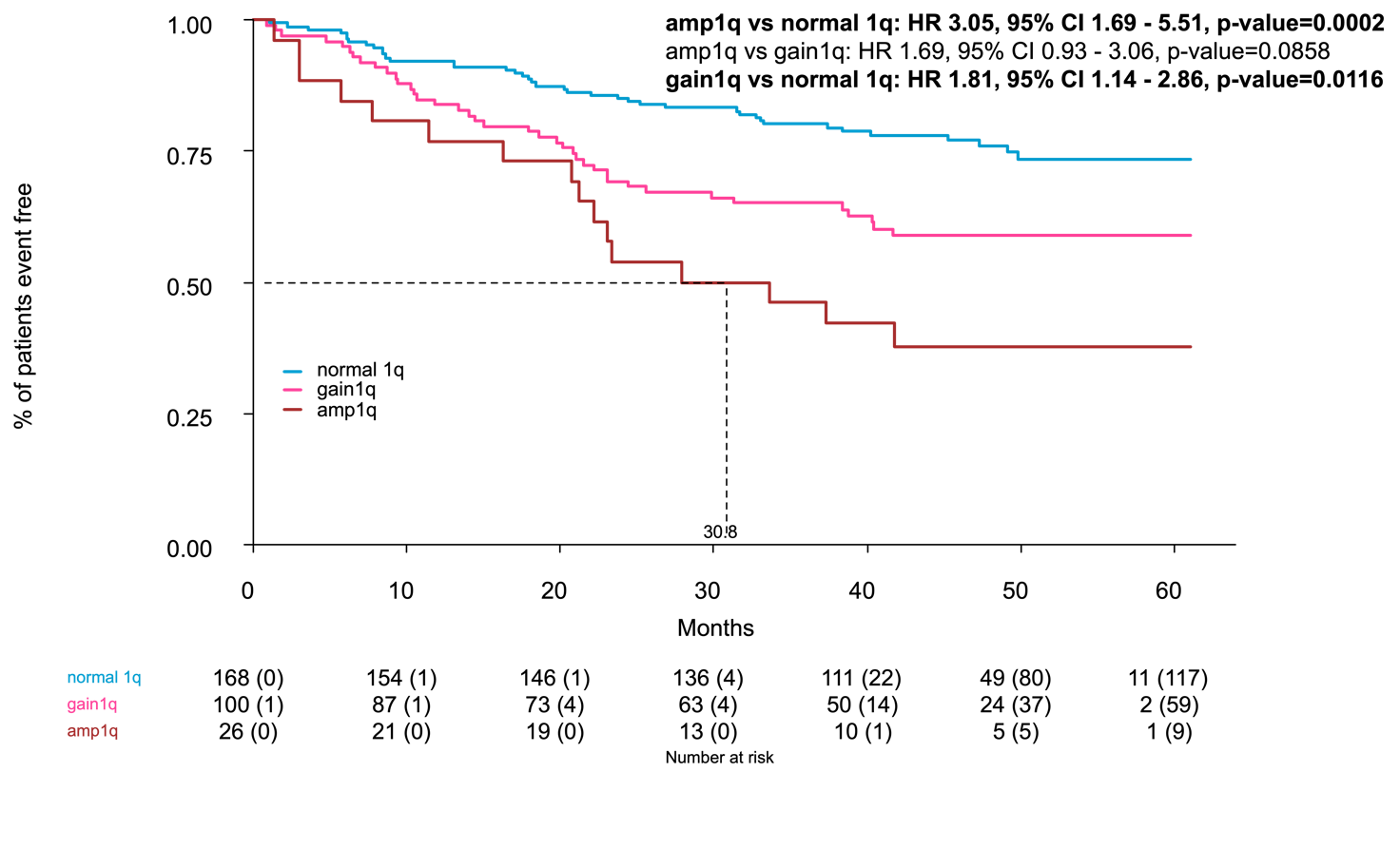


Figure S3. Subgroup analysis of PFS comparing Amp1q vs Normal1q (A); Gain1q vs Normal1q (B) and Amp1q vs Gain1q (C).

**Figure S4.** Progression-free survival from first randomization according to 1q subgroups and Minimal Residual disease (MRD) status at 10^-5^ by Multiparameter flow cytometry (MFC).

(A) Premaintenance MRD positive by MFC in ITT; (B) Premaintenance MRD negative by MFC in ITT; (C) 1-year sustained MRD negative by MFC in ITT.

Figure S5. Principal Component Analysis of NDMM patients with or without concomitant t(4;14) in Normal1q, Gain1q and Amp1q group show an interaction of t(4;14) with the RNAseq data of Amp1q patients (A). Separation of patients without concomitant t(4;14) in 1q-defined groups according to RNAseq data is shown in panel B. Selected genes deregulated in Gain1q and Amp1q patients compared to normal 1q are shown in panel C.


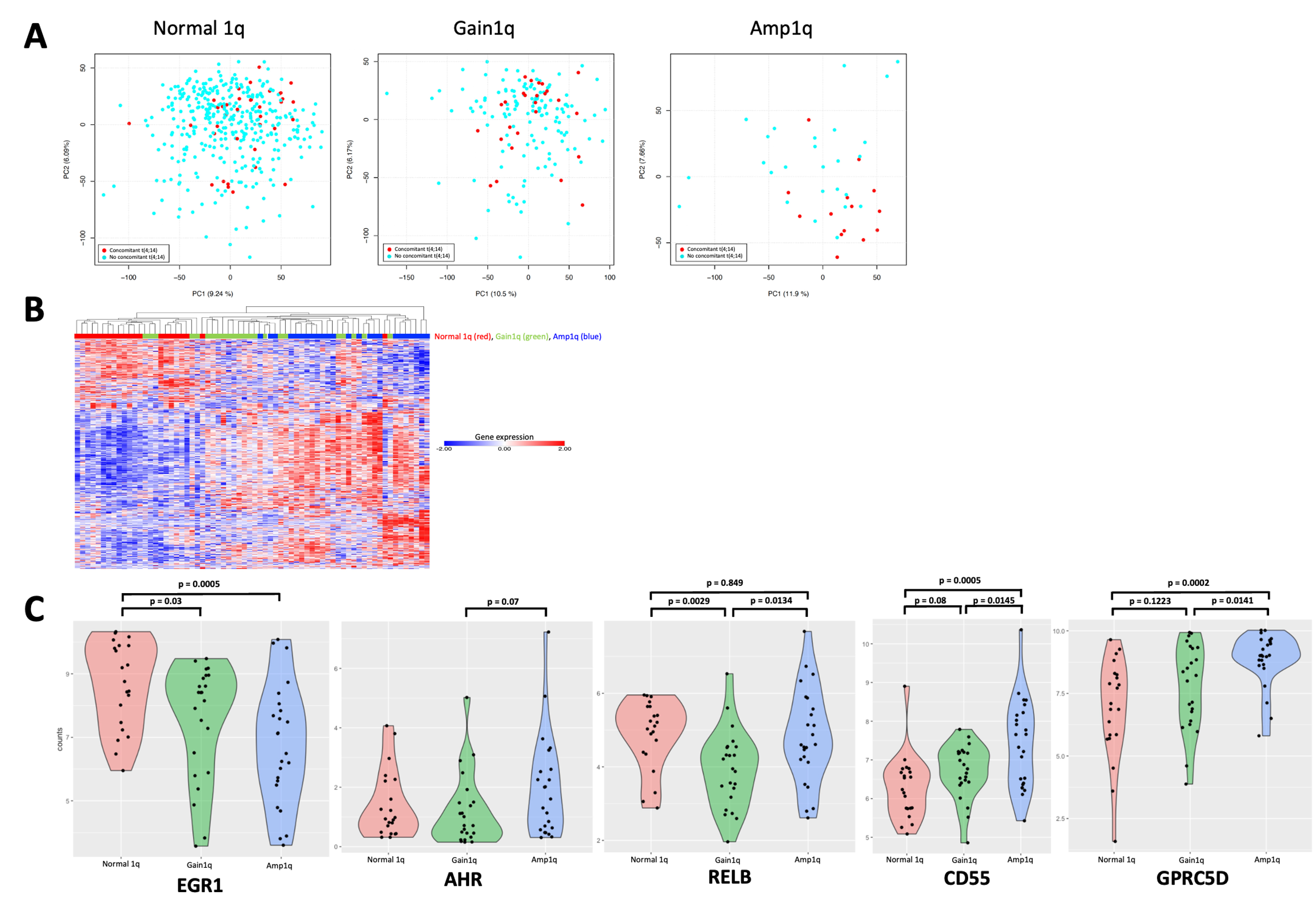


1. Jiang H, Lei R, Ding SW, Zhu S. Skewer: a fast and accurate adapter trimmer for next-generation sequencing paired-end reads. BMC Bioinformatics. 2014 Dec 12;15(1):182.

2. Dobin A, Davis CA, Schlesinger F, Drenkow J, Zaleski C, Jha S, et al. STAR: ultrafast universal RNA-seq aligner. Bioinformatics. 2013 Jan 1;29(1):15–21.

3. Li B, Dewey CN. RSEM: accurate transcript quantification from RNA-Seq data with or without a reference genome. BMC Bioinformatics. 2011 Dec 4;12(1):323.

4. Beccuti M, Cordero F, Arigoni M, Panero R, Amparore EG, Donatelli S, et al. SeqBox: RNAseq/ChIPseq reproducible analysis on a consumer game computer. Bioinformatics. 2018 Mar 1;34(5):871–2.

5. Robinson MD, McCarthy DJ, Smyth GK. edgeR: a Bioconductor package for differential expression analysis of digital gene expression data. Bioinformatics. 2010 Jan 1;26(1):139–40.

6. Edgar R. Gene Expression Omnibus: NCBI gene expression and hybridization array data repository. Nucleic Acids Res. 2002 Jan 1;30(1):207–10.
